# Transcutaneous vagus nerve stimulation boosts evidence accumulation during perceptual decision making

**DOI:** 10.1016/j.isci.2026.117748

**Published:** 2026-10-08

**Authors:** Shiyong Su, Thomas Vanvoorden, Pierre Le Denmat, Alexandre Zénon, Julie Duque

**Affiliations:** 1 State Key Laboratory of Mechanical System and Vibration, School of Mechanical Engineering, Shanghai Jiao Tong University, Shanghai 200240, China; 2 Shanghai Key Laboratory of Intelligent Robotics, Shanghai Jiao Tong University, Shanghai 200240, China; 3 Meta Robotics Institute, Shanghai Jiao Tong University, Shanghai 200240, China; 4 Cognition and Actions Lab, Institute of Neuroscience, UCLouvain, Brussels, Belgium; 5 Department of Clinical Neuroscience, Karolinska Institute, Stockholm, Sweden; 6 Institut de Neurosciences Cognitives et Intégratives d’Aquitaine, Bordeaux, France

**Keywords:** pupil-linked arousal, transcutaneous vagus nerve stimulation, decision-making, random dot motion, drift diffusion model, gain modulation, attention, evidence accumulation, cognitive control

## Abstract

Ascending arousal systems, including the locus coeruleus-norepinephrine (LC-NE) system, may shape perceptual decision-making by enhancing sensory processing (“gain”) or accelerating response commitment (“urgency”). We examined how arousal-related neuromodulatory activity shapes decisions and whether its effects depend on arousal state. We tested 62 participants in three groups, receiving transcutaneous vagus nerve stimulation (tVNS) at three timings during a random dot motion task. tVNS increased pupil-linked arousal, with early stimulation producing more sustained dilation into decision-making. Behaviorally, tVNS selectively improved accuracy under initially low performance without affecting response times. Drift diffusion modeling linked this improvement to increased drift rate, consistent with more efficient evidence accumulation. These effects occurred across groups but were most pronounced with early stimulation. Our findings support a gain-related enhancement of evidence accumulation under tVNS and suggest that pupil-linked arousal, potentially involving LC-NE among other ascending arousal pathways, may help stabilize attentional engagement when endogenous control is suboptimal.

## Highlights

• tVNS increases pupil-linked arousal across stimulation timings
• tVNS improves decision accuracy without changes in decision speed
• tVNS selectively improves accuracy in conditions with initially lower accuracy
• Higher drift rates support gain-related enhancement in evidence accumulation under tVNS

## Graphical abstract

**Figure:**
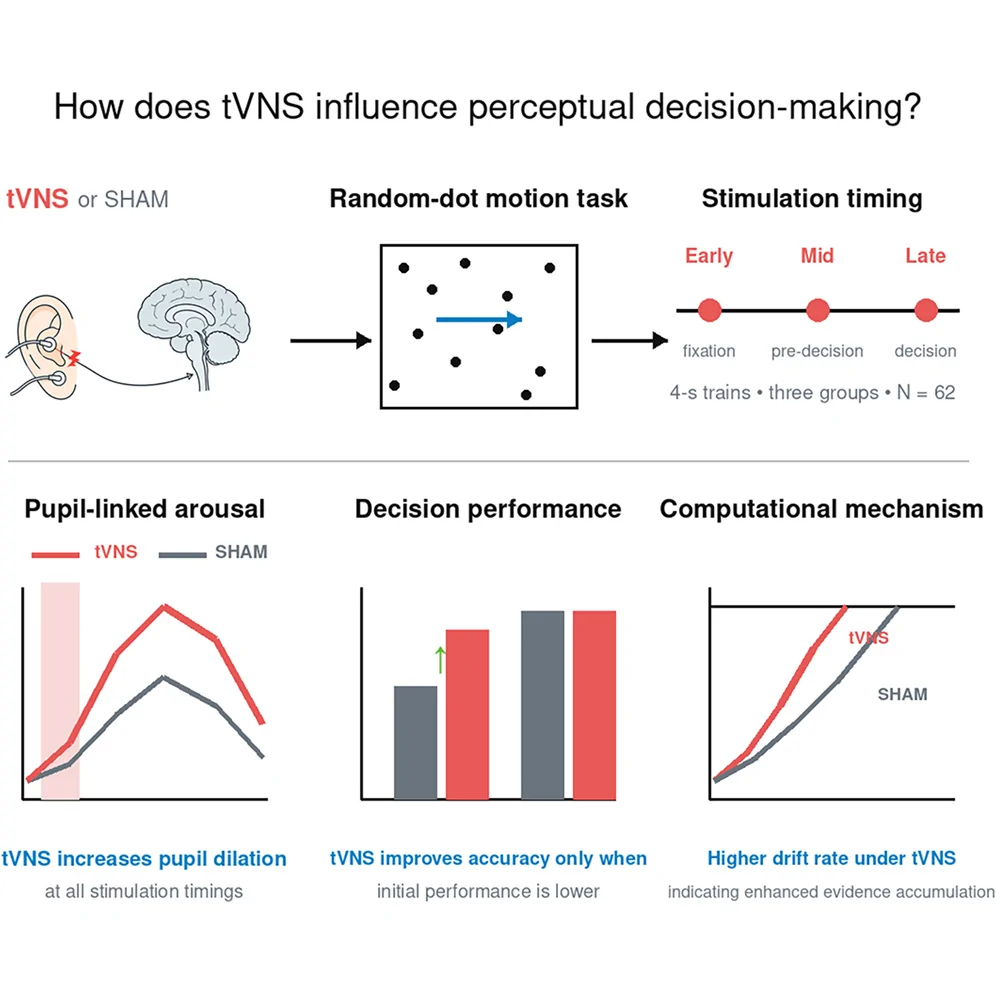

## Teaser

Natural sciences; Biological sciences; Cognitive neuroscience

## Introduction

The ascending arousal system consists of brainstem and subcortical neuromodulatory pathways that regulate global brain state and support a wide range of adaptive behaviors. A prominent component of this system is the locus coeruleus-norepinephrine (LC-NE) pathway.^1^^,^^2^^,^^3^^,^^4^^,^^5^^,^^6^ The LC is a small brainstem nucleus and the principal source of norepinephrine in the brain, with widespread projections to cortical and subcortical regions.^1^^,^^3^ Through these projections, the LC-NE system contributes to arousal regulation, attentional engagement, and behavioral flexibility.^3^ Recent work has increasingly implicated arousal-related neuromodulatory systems, including the LC-NE system, in perceptual decision-making, which requires the accumulation of sensory evidence to select the most appropriate motor response.^7^^,^^8^ However, their precise role in this context remains debated. A dominant view from animal studies proposes that heightened arousal-related neuromodulatory activity may improve decision accuracy by enhancing the signal-to-noise ratio of sensory processing, a mechanism known as “gain” modulation.^1^^,^^9^^,^^10^^,^^11^^,^^12^^,^^13^ In contrast, recent human studies have advanced an alternative “urgency” hypothesis, suggesting that increased arousal-related neuromodulatory activity accelerates decision commitment and action release, potentially at the cost of accuracy.^14^^,^^15^^,^^16^ Yet, direct empirical evidence on perceptual decision-making in humans that can clearly distinguish between these competing views remains scarce.

One reason for the limited empirical support in humans for either hypothesis is that most studies have relied on correlational approaches, primarily using pupil size as an indirect index of arousal engagement,^16^^,^^17^ consistent with evidence showing that direct activation of brainstem arousal nuclei, including LC, evokes pupil dilation.^18^ While informative, such methods do not allow for definitive conclusions about the causal role of arousal-related neuromodulatory activity in perceptual decision-making. In recent years, however, transcutaneous vagus nerve stimulation (tVNS) has emerged as a promising non-invasive technique to causally probe arousal function. This approach involves stimulation of the auricular branch of the vagus nerve at the level of the ear, which has been associated with increased arousal engagement via afferent projections to the nucleus tractus solitarii (NTS), which in turn projects to the LC among other targets.^19^ Beyond its effect on arousal-related physiology, a growing body of evidence suggests that tVNS can modulate cognitive performance through gain-related mechanisms, with its effect depending on current processing demands rather than reflecting a nonspecific increase in arousal.^20^ Consistent with this view, previous human studies have shown selective effects of tVNS across several cognitive control domains, including response selection,^21^ inhibitory control,^22^ conflict monitoring,^23^ and working-memory gating.^24^ More closely related to perceptual function, vagus nerve stimulation has been shown to enhance sensory processing in rodents,^25^ while non-invasive cervical vagus nerve stimulation has been associated with improved perceptual performance in humans.^26^ Using a different tVNS protocol in which stimulation was delivered as brief 4-s online trains on each trial of a random dot motion task, we previously reported initial observations in humans that were consistent with the “gain” theory.^27^ Specifically, online tVNS was associated with improved decision accuracy without altering response speed. Notably, this improvement emerged only after errors, when accuracy in that condition had declined, effectively restoring performance to average levels, while having no measurable effect when performance was already adequate. This state-dependent pattern is consistent with recent findings showing greater sensory benefits of vagus nerve stimulation in individuals with lower baseline sensory performance,^26^ and align with the idea that arousal-related neuromodulatory activity helps optimize the brain’s limited energetic resources by selectively enhancing performance when it benefits goal-directed behavior.^28^^,^^29^^,^^30^ However, while these findings provided initial support for the gain-related modulation of decision accuracy, the underlying mechanisms through which tVNS influences perceptual decision-making remain unclear.

Pupil-linked arousal is known to fluctuate with task engagement progress, rising from baseline levels at rest to higher levels during task performance.^1^^,^^4^^,^^16^^,^^31^ In our previous study, we further observed that the dynamics of tVNS-induced pupil response differ across these states.^27^ Specifically, when tVNS was applied at rest, the evoked pupil dilation rapidly returned to baseline once stimulation ceased. In contrast, when stimulation was delivered during task performance, the tVNS-induced pupil dilation persisted not only throughout stimulation but also beyond its offset, even until the task ended. This contrast between rest and task engagement suggests that tVNS effects on pupil dilation may vary not only across states but also across phases of arousal engagement within a task, potentially leading to distinct behavioral outcomes, consistent with the arousal’s inverted-U-shaped relationship to performance.^1^^,^^29^

The present study therefore pursued two complementary goals. First, using 4-s online tVNS trains delivered within individual trials, we aimed to clarify the computational mechanism through which tVNS influences perceptual decision-making, specifically whether its effects are more consistent with gain-related enhancement of evidence accumulation or urgency-related response commitment. Second, we sought to determine whether the behavioral and computational effects of tVNS depend on the timing of stimulation within the decision process, and whether these variations are associated with differences in pupil-linked arousal engagement. To address these questions, we tested 62 healthy participants who performed the random dot motion task while receiving brief 4-s trains of tVNS or SHAM stimulation in separate blocks, with pupil size continuously recorded as a physiological proxy of arousal engagement. Participants were instructed to respond as accurately and quickly as possible, but received feedback only on accuracy, not speed, at the end of each trial. We hypothesized that tVNS would selectively enhance decision accuracy, particularly in conditions of suboptimal performance, as reflected by higher error rates in the SHAM blocks, and that this improvement would be associated with an increase in drift rate, indicating more efficient model-derived evidence accumulation, consistent with the gain modulation framework. Moreover, to probe potential phase-dependent effects, participants were assigned to one of three groups that received tVNS or SHAM stimulation at distinct time points within each trial: well before, immediately before, or during decision-making. These timings allowed us to assess whether stimulation timing and the corresponding modulation of pupil-linked arousal would differentially influence decision behavior and its computational mechanisms.

## Results

Sixty-two participants performed a random dot motion (RDM) task under tVNS and SHAM stimulation conditions (see Figures 1A and 1B and STAR Methods for more details). The RDM task required participants to discriminate the direction of coherently moving dots within a random display, as fast and accurately as possible. Reaction time (RT) and accuracy served as primary behavioral outcome measures, while pupil size was considered to assess arousal engagement during the task in tVNS and SHAM blocks. Participants were assigned to one of three groups based on the timing of the 4-s tVNS/SHAM stimulation within each trial, corresponding to distinct phases of arousal engagement as informed by our previous study (Su, Vanvoorden et al., 2024): Group_Early_ (*n* = 20) received stimulation early in the trial, during the Fixation phase, when arousal engagement was presumed to be at baseline. Group_Mid_ (*n* = 21), whose data were included in our previous study, received stimulation at a middle period spanning the Fixation and Pre-decision phases, aiming to target the arousal-related neuromodulatory system as its activity began to rise. Group_Late_ (*n* = 21) received stimulation late in the trial, spanning the pre-decision and decision phases, when arousal engagement was reaching its task-related peak. All participants received tVNS and SHAM stimulation in alternating blocks, allowing for within-subject comparisons in each group.

**Figure 1:**
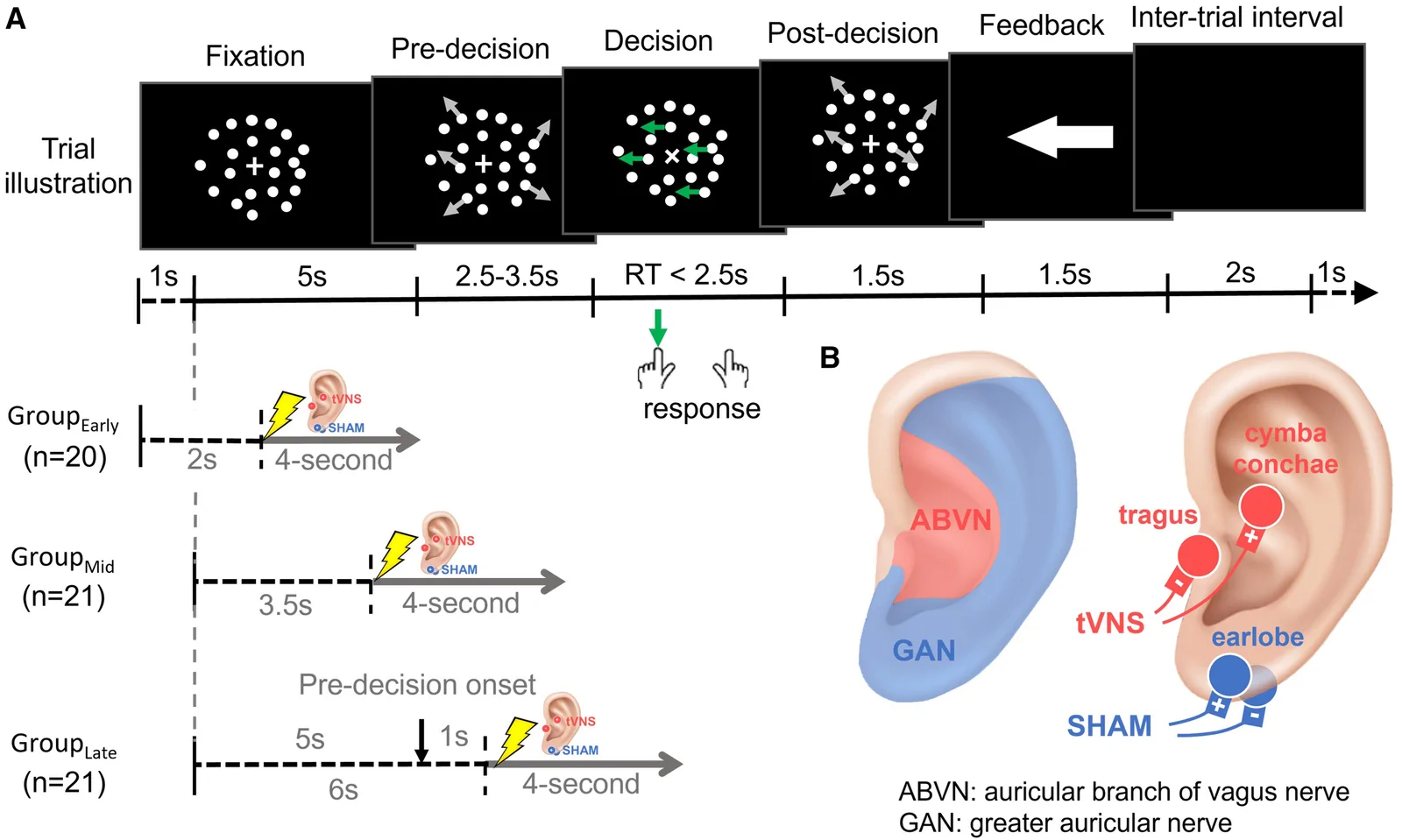
Experimental design (A) Trial illustration in the random dot motion (RDM) task. Each trial consisted of a Fixation phase (6s in Group_Early_, 5 s in Group_Mid_ and Group_Late_), a Pre-decision phase with randomly moving dots (2.5–3.5s), and a Decision phase in which the central cross changed from the “+” to an “x” and a proportion of dots moved coherently to the left or right (left here). Participants reported motion direction with a left or right key press as quickly and accurately as possible within 2.5s. The motion then returned to random (Post-decision phase, 1.5 s), followed by visual feedback (1.5 s). Group_Early_ included a 1-s longer Fixation phase to ensure the full 4-s stimulation train occurred entirely within the Fixation phase while preserving a stimulation-free pupil baseline window. To maintain equivalent overall trial duration across groups, the inter-trial interval was reduced by 1s in Group_Early_ (2s instead of 3s). A 4-s train of tVNS/SHAM was applied during the task. Participants were either part of Group_Early_ (*n* = 20), Group_Mid_ (*n* = 21), or Group_Late_ (*n* = 21), depending on the timing of tVNS/SHAM stimulation. In Group_Early_, participants received tVNS/SHAM stimulation early in the trial, 2s after the onset of the Fixation phase. In Group_Mid_, participants received tVNS/SHAM stimulation at a middle time point, 3.5s after the 5s Fixation phase onset, thus covering the Fixation and Pre-decision phases. In Group_Late_, participants received stimulation late in the trial, 1s after the onset of the Pre-Decision phase, thereby spanning the Pre-decision and Decision phases. (B) tVNS setup. Electrodes were placed on the cymba conchae and tragus of the left ear in the tVNS condition, allowing the generation of an electric field covering the area innervated by the auricular branch of the vagus nerve. Electrodes were placed on the left earlobe in the SHAM condition, covering the area of the greater auricular nerve.

To account for individual differences in visual perception, the coherence level (c’) of moving dots in the RDM task was adjusted at the beginning of the experiment for each participant to achieve approximately 70% baseline accuracy. This calibration procedure resulted in overall similar coherence levels across the three groups (see Figure S1A), with accuracy in SHAM blocks averaging 73.86% (see Figure S1B; Table S1 for more details on statistics).

### tVNS systematically increased pupil dilation through decision-making, regardless of its application time

Pupil size was continuously recorded throughout the RDM task. For all groups, pupil size was normalized within each participant using a *Z* score and was then segmented into trials aligned either to the onset of stimulation (stimulation-aligned pupil response) or to the onset of coherent dot motion (decision onset, decision-aligned pupil response). Pupil dilation was quantified as a change from pupil baseline, with the latter always computed over a 1-s window starting 1s after Fixation onset (see STAR Methods and Figures 2A–2C, left image).

**Figure 2:**
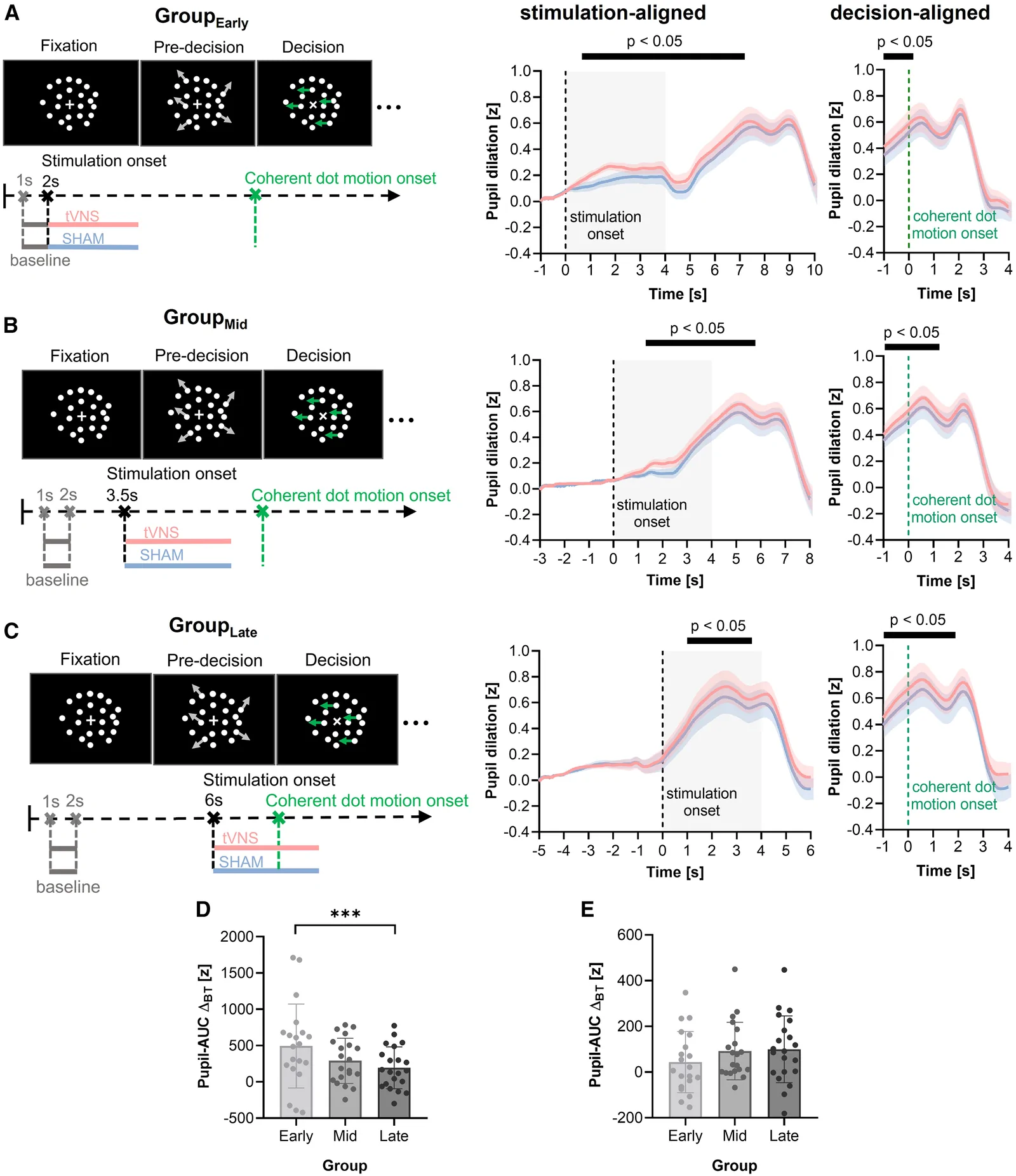
Pupil response in the RDM task (A) Pupil dilation in Group_Early_. The graphs display pupil dilation aligned to stimulation onset (middle image) and coherent dot motion onset (right image) in Group_Early_, separated for tVNS (red) and SHAM (blue) blocks. Shaded areas around the traces represent ±SEM. The gray transparent rectangle indicates the stimulation period. Black horizontal lines above the traces indicate periods with significant differences between tVNS and SHAM blocks, identified using paired *t* tests with cluster-based permutation correction (*p* < 0.05). Pupil dilation was significantly greater in tVNS than SHAM blocks from 0.62 s to 7.23 s after stimulation onset, and up to 0.16 s after motion onset. (B) Pupil dilation in Group_Mid_. Same format as in A, for Group_Mid_. Significant tVNS-induced pupil dilation was observed from 1.21 s to 5.85 s post-stimulation onset, and up to 1.24 s post-coherent motion onset. (C) Pupil dilation in Group_Late_. Same format as in A, for Group_Late_. Pupil dilation was significantly greater in tVNS than SHAM blocks from 0.98 s to 3.64 s after stimulation onset, and up to 1.85 s after coherent motion onset. (D) Stimulation-aligned Pupil-AUC Δ_BT_. This graph shows the overall effect of tVNS on pupil dilation, expressed as the Pupil-AUC Δ_BT_, which corresponds to the area between the tVNS and SHAM curves, computed over the period showing significant tVNS effects in each group (indicated by the black horizontal lines above the traces in the middle image of A-C). Each dot represents one participant from Group_Early_ (light gray), Group_Mid_ (medium gray), or Group_Late_ (dark gray); bars indicate group means ± SEM. Note the greater Pupil-AUC Δ_BT_ in Group_Early_ than in Group_Late_. (E) Decision-aligned Pupil-AUC ΔBT. Same format as in D, but here the Pupil-AUC Δ_BT_ was computed from the onset of coherent dot motion to the participant’s response. Note the comparable Pupil-AUC Δ_BT_ during decision-making across groups. ∗∗∗: *p* < 0.001.

Across groups, pupil dilation consistently increased throughout the RDM task trials, peaking approximately 0.5s after the onset of coherent dot motion before gradually returning to baseline (see Figures 2A–2C). The dynamics of pupil dilation under tVNS and SHAM conditions across groups are summarized in Table S2. Importantly, all groups exhibited significant differences in stimulation-aligned pupil dilation between tVNS and SHAM conditions (Figures 2A–2C, middle image), with paired *t* tests using cluster-based permutations revealing significantly greater pupil dilation under tVNS compared to SHAM (*p* < 0.05), from 0.62s to 7.23s in Group_Early_, from 1.21s to 5.85s in Group_Mid_, and from 0.98s to 3.64s in Group_Late_. Note that a slight increase in pupil dilation was already observable immediately preceding stimulation onset (Figure 2A, middle image), which could reflect learned anticipation of the stimulation over the course of the session. To address this specifically, we compared this pre-stimulation pupil dilation during a [-0.5 0]s window between the first and second halves of the session using a linear mixed effects model including Block-Type (tVNS, SHAM), Group (Early, Mid, Late), and Session-Half (first, second) as factors. However, the analysis only revealed a non-significant tendency for pupil dilation to decrease rather than increase over the course of the experiment (main effect of Session-Half: *F* = 3.225, *p* = 0.073), with no interaction involving Block-Type (F < 1.014, *p* > 0.363) and no significant three-way interaction (*F* = 0.052, *p* = 0.949). Hence, this slight pre-stimulation pupil increase is unlikely to reflect learned anticipation of stimulation onset but may indicate a transient increase in attentional engagement at the beginning of the task, when the Fixation display with static dots appeared (see Figure S2). In contrast, the post-stimulation analysis indicated larger pupil dilation under tVNS than under sham, emerging shortly after stimulation onset (between 0.62s and 1.21s), and sustained until approximately the time of the participant’s response. Hence, the duration of the tVNS effect varied across groups, appearing longest in Group_Early_ and shortest in Group_Late_. To assess the resulting difference in the overall magnitude of the tVNS-induced pupil dilation across groups, we computed the cumulative difference in pupil dilation between tVNS and SHAM conditions over the period showing a significant tVNS effect. Specifically, we calculated the area under the curve (AUC) of pupil dilation traces aligned to stimulation onset for each subject, separately for each Block-Type (BT: tVNS and SHAM). The SHAM AUC was then subtracted from the tVNS AUC to derive a delta (Δ) measure reflecting tVNS-induced pupil dilation, referred to as stimulation-aligned Pupil-AUC Δ_BT_. The ANOVA run on this measure revealed a significant main effect of Group (F(2, 58) = 6.901, *p* = 0.002), indicating a greater Pupil-AUC Δ_BT_ with early compared to late tVNS timing (see Figure 2D, Group_Early_ vs. Group_Late_: *t* = 3.681, *p*_*tukey*_ = 0.001).

When aligning pupil dilation to coherent dot motion onset (decision-aligned pupil dilation), cluster-based permutation tests also revealed significantly larger pupil dilation under tVNS than SHAM in all groups (Figures 2A–2C, right image): from -1s to 0.16s in Group_Early_, -1s–1.24s in Group_Mid_, and -1s–1.85s in Group_Late_ (all *p* < 0.05). Here too, we extracted a similar decision-aligned Pupil-AUC Δ_BT_ to assess the overall tVNS-induced pupil dilation specifically during the decision phase, thus starting from the onset of coherent dot motion to the participants’ response. When restricting our analysis to this period, ANOVA revealed a comparable Pupil-AUC Δ_BT_ across groups, as confirmed by the absence of a group effect (F(2, 58) = 0.793, *p* = 0.458, Figure 2E). Our results therefore provide strong evidence that, regardless of the timing of its application, the effect of tVNS tended to subside around the time of the response, even when applied early in the trial in Group_Early_. Hence, early stimulation (Group_Early_) led to a longer-lasting effect on pupil dilation such that the magnitude of the effect during decision-making was ultimately comparable across the three groups.

### tVNS did not alter pupil baseline but the latter was increased following errors

Analyses on a subset of subjects from our previous study (Group_Mid_, *n* = 20) suggested that using sufficiently long inter-stimulation intervals (∼11 s) allows the tVNS effect on pupil to dissipate before the next trial begins (Su, Vanvoorden et al. 2025). That study also showed a larger pupil baseline following errors compared to correct trials. To verify this pattern in the present, larger dataset (*n* = 62), we first analyzed pupil baseline separately within each group, with Block-Type (tVNS, SHAM) and Trial-History (post-correct, post-error) as within-subject factors. Across all groups, the results revealed a consistent pattern: there was no significant main effect of Block-Type (all F < 2.350, all *p* > 0.125), but a reliable main effect of Trial-History, with higher pupil baseline following errors compared to correct trials (all F > 23.144, all *p* < 0.001). This effect occurred irrespective of Block-Type, as indicated by the nonsignificant Block-Type × Trial-History interaction (all F < 1.910, all *p* > 0.167). Given this consistency across groups, data were pooled across all participants for a unified linear mixed-effects model. This model confirmed the absence of a Block-Type effect on pupil baseline (F(1, 23.8) = 0.529, *p* = 0.474, see Figure S3A) and again revealed a robust main effect of Trial-History, with larger pupil baseline following errors than correct trials (F(1, 17.3) = 86.666, *p* < 0.001, see Figure S3B). The Block-Type × Trial-History interaction remained nonsignificant (F(1, 17069.4) = 1.457, *p* = 0.227). Detailed statistical results, including effect-size estimates and confidence intervals, are provided in Table S3. These results confirm that tVNS did not alter pupil baseline, even when it was applied later on in the trials of Group_Late_, while baseline levels were consistently higher following errors, replicating and extending previous findings.

### tVNS enhanced accuracy irrespective of its application time in the RDM task and without affecting RTs

To determine whether the activation of pupil-linked arousal by tVNS influences behavior and whether its effects depend on the timing of its application, accuracy and RTs were analyzed using generalized and linear mixed-effects models, respectively. Notably, accuracy was higher in tVNS blocks (Group_Early_ = 73.4 ± 1.5%, Group_Mid_ = 77.8 ± 1.8%, Group_Late_ = 76.0 ± 1.6%) compared to SHAM blocks (Group_Early_ = 70.5 ± 1.6%, Group_Mid_ = 75.4 ± 1.8%, Group_Late_ = 75.6 ± 1.9%) in all groups (see Figure 3A), as confirmed by a significant main effect of Block-Type (χ^2^ (1) = 8.77, *p* = 0.003) with no Block-Type × Group interaction (χ^2^ (2) = 1.145, *p* = 0.564) in the generalized mixed-effects model. Despite the relatively modest effect size, the improvement in accuracy under tVNS was consistent across participants in Group_Early_ (17/20) and Group_Mid_ (17/21), and still evident in a majority of Group_Late_ participants (13/21). In contrast, the linear mixed-effects model on RTs did not reveal any effect of Block-Type (F < 1.874, *p* > 0.154). As such, RTs in all groups were comparable in tVNS blocks (Group_Early_ = 1.105 ± 0.061s, Group_Mid_ = 1.314 ± 0.054s, Group_Late_ = 1.247 ± 0.045s) and SHAM blocks (Group_Early_ = 1.123 ± 0.057s, Group_Mid_ = 1.322 ± 0.049s, Group_Late_ = 1.238 ± 0.046s; see Figure 3B). However, we observed a main effect of Group, with participants in Group_Mid_ exhibiting the slowest overall RTs, while those in Group_Early_ responded the fastest (F(2, 15800.4) = 327.29, *p* < 0.001), regardless of the Block-Type (Block-Type × Group interaction: F(2, 13551) = 1.175, *p* = 0.309).

**Figure 3:**
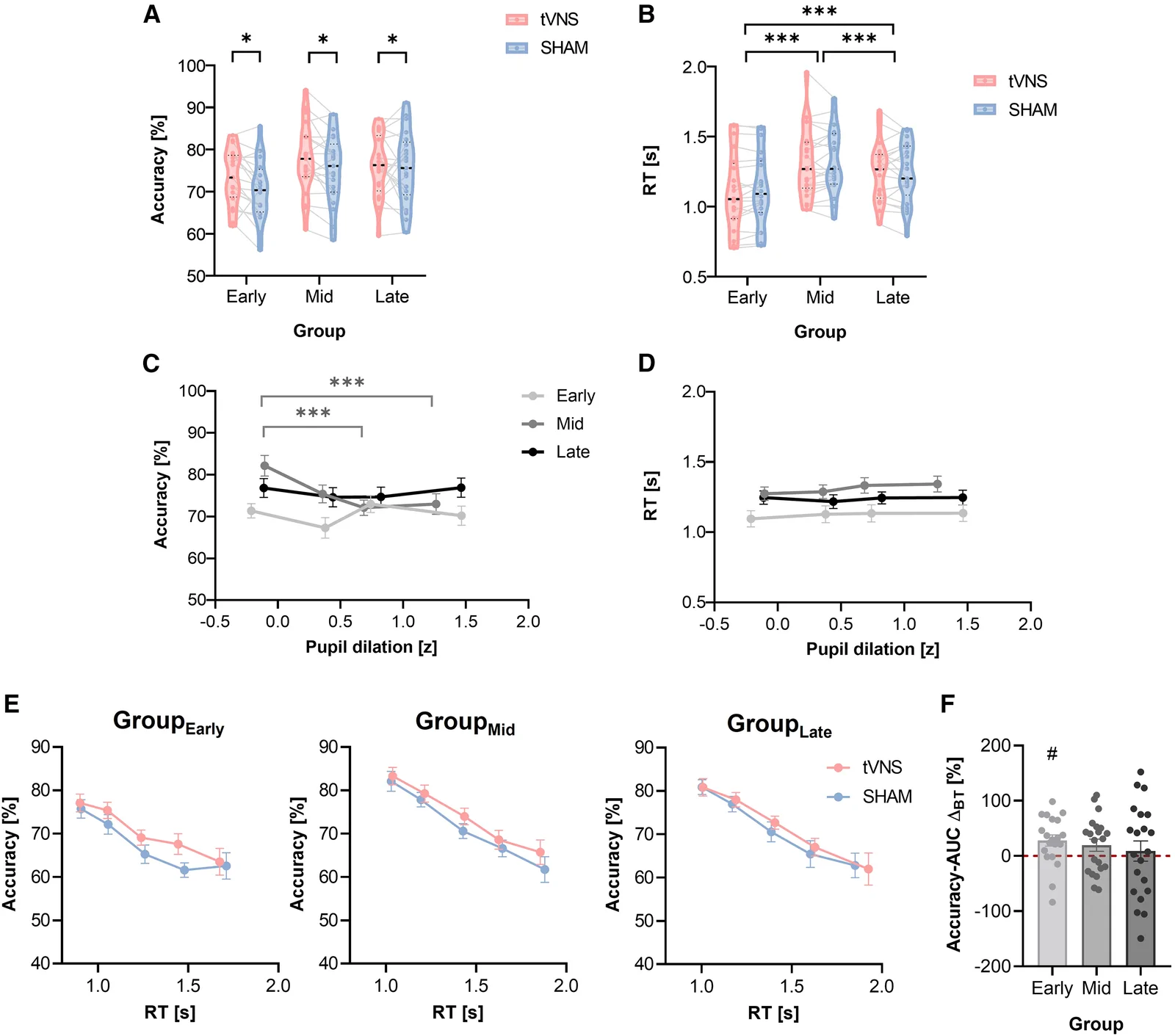
Accuracy and reaction times (RT) in the RDM task (A) General tVNS effect on accuracy across groups. The data features median values as black dotted lines, distributions as violin plots, and individual data as thin gray lines, separated for tVNS (red) and SHAM (blue) blocks in each group. Note the greater accuracy in tVNS compared to SHAM blocks in all groups. (B) General tVNS effect on RT across groups. Same format as in A. Note comparable RTs in tVNS and SHAM blocks in all groups. (C) Effect of pupil dilation on accuracy in SHAM blocks. Data are displayed as Mean ± SEM across pupil dilation bins for Group_Early_ (light gray), Group_Mid_ (medium gray), and Group_Late_ (dark gray) groups. Accuracy remained stable across pupil dilation bins in Group_Early_ and Groups_Late_, whereas a trend toward reduced accuracy with greater pupil dilation was observed in Group_Mid_. (D) Effect of pupil dilation on RT in SHAM blocks. Same format as in C. RTs remained stable across pupil dilation bins in all groups. (E) tVNS effect on conditional accuracy curves across groups. Accuracy is plotted as a function of RT, with each data point representing the mean ± SEM of accuracy within RT bins. In all groups, the conditional accuracy curve under tVNS (red) was shifted upward relative to SHAM (blue), indicating an improved speed-accuracy tradeoff with greater accuracy at comparable RTs. (F) Accuracy-AUC Δ_BT_ across groups. Bars and individual dots represent the mean ± SEM of delta accuracy (tVNS minus SHAM) integrated across all RT bins. While there was no significant difference in Accuracy-AUC Δ_BT_ across groups, only Group_Early_ showed values significantly above zero. ∗: *p* < 0.05. ∗∗∗: *p* < 0.001. #: *p* < 0.05.

Following these findings, we examined whether the accuracy enhancement observed under tVNS could be explained by optical consequences of a physically larger pupil size. To control for this potential bias, we used a generalized mixed-effects model to test the relationship between accuracy and decision-aligned pupil dilation in SHAM blocks. The analysis revealed a significant Pupil dilation × Group interaction on accuracy (χ^2^ (2) = 7.29, *p* = 0.026), indicating that, if anything, accuracy tended to decrease (rather than increase) with greater pupil size (Figure 3C), an effect only significant in Group_Mid_ (bin1 VS. bin3: *z* = 4.512, *p*_*tukey*_ < 0.001; bin1 VS. bin4: *z* = 4.046, *p*_*tukey*_ = 0.003). A linear mixed-effects model did not reveal any effects of pupil dilation on RTs in SHAM blocks (F < 3.05, *p* > 0.07; Figure 3D). Furthermore, the magnitude of tVNS-induced pupil dilation was not correlated with the behavioral effect of tVNS on accuracy across participants either (Figure S4). Therefore, the behavioral effects observed in tVNS blocks are unlikely to result from physically larger pupils in this condition. Instead, tVNS appeared to enhance subjects' decision accuracy without compromising RTs, as further illustrated by an upward shift in the conditional accuracy curve of the three groups (see Figure 3E). To assess the overall impact of tVNS on decision accuracy across the RT distribution, we computed the difference in the area under the conditional accuracy curve (AUC) between each Block-Type (BT: tVNS and SHAM). Specifically, for each subject, we first calculated the delta of accuracy between tVNS and SHAM blocks within each RT bin, based on the average RTs obtained in the two types of blocks. These values were then integrated across the RT range using the trapezoidal rule to obtain a single summary measure, referred to as Accuracy-AUC Δ_BT_. This metric captures the overall magnitude of tVNS-induced accuracy changes over the temporal profile of decisions. A one-way ANOVA revealed no significant difference in Accuracy-AUC Δ_BT_ across groups (F(2, 59) = 0.487, *p* = 0.617; see Figure 3F), suggesting that the overall effect of tVNS on shifting the conditional accuracy curve was comparable across groups with different tVNS timings. However, one-sample *t* tests against zero indicated that only Group_Early_ showed a significantly positive Accuracy-AUC Δ_BT_ (t = 2.769, *p* = 0.012), while Group_Mid_ (t = 1.778, *p* = 0.091) and Group_Late_ (t = 1.602, *p* = 0.121) did not reach statistical significance.

### tVNS improved accuracy in conditions with initially lower accuracy

The generalized mixed-effects model on accuracy also considered Trial-History (TH: post-correct, post-error) as a fixed effect and incorporated “Accuracy Δ_TH_” in SHAM blocks as a covariate. This covariate represents each participant’s overall delta of accuracy between trials following errors compared to trials following correct trials (post-error minus post-correct) in SHAM blocks (see Figure 4A, blue dots) and was used to identify which condition (post-error or post-correct) yielded the lowest accuracy for each individual in the absence of tVNS. A negative Accuracy Δ_TH_ in SHAM blocks indicates lower accuracy in post-error compared to post-correct trials, whereas a positive value reflects the opposite pattern, with lower accuracy in post-correct trials. Notably, there was a significant Block-Type × Trial-History × Accuracy Δ_TH_ interaction (χ^2^ (1) = 50.316, *p* < 0.001); the quadruple interaction with Group was not significant (χ^2^ (2) = 1.311, *p* = 0.519). To explore this interaction, participants were separated into two fairly balanced clusters depending on the sign of their Accuracy Δ_TH_ values in SHAM blocks (see Figure 4A): 32 participants showed negative values, indicating lower accuracy (LowACC) in post-error (PE) trials (LowACC-PE cluster), while 30 participants exhibited positive values, indicating lower accuracy in post-correct (PC) trials (LowACC-PC cluster). A comparison of coherence values (c’) showed a trend toward higher coherence (F(1, 60) = 3.693, *p* = 0.059) in the LowACC-PE cluster (0.237 ± 0.027) than in the LowACC-PC cluster (0.172 ± 0.019), consistent with our previous finding that higher coherence leads to poorer performance after errors in our task (Su, Vanvoorden et al. 2025). As expected by definition, within SHAM blocks, participants in the LowACC-PE cluster exhibited significantly lower accuracy in post-error compared to post-correct trials (*z* = 8.246, *p*_*tukey*_ < 0.001; Figure 4B, left image), while participants in the LowACC-PC cluster showed lower accuracy in post-correct than in post-error trials (*z* = −3.480, *p*_*tukey*_ = 0.003; see Figure 4B, right image). More interestingly, tVNS selectively enhanced accuracy in post-error trials of LowACC-PE participants (post-correct: *z* = −2.018, *p*_*tukey*_ = 0.181; post-error: *z* = 4.687, *p*_*tukey*_ < 0.001), while it selectively improved accuracy in post-correct trials of LowACC-PC participants (post-correct: *z* = 4.055, *p*_*tukey*_ < 0.001; post-error: *z* = −1.053, *p*_*tukey*_ = 0.718). Detailed statistical results including effect-size estimates and confidence intervals are provided in Table S4. Hence, the tVNS train enhanced accuracy in both participant clusters, but did so in different conditions, specifically in the one where they initially showed lower accuracy in SHAM blocks (LowACC trials, surrounded by red rectangles in Figure 4B). Consequently, in tVNS blocks, the Accuracy Δ_TH_ became less negative for LowACC-PE participants and less positive for LowACC-PC participants (see Figure 4A, red dots).

**Figure 4:**
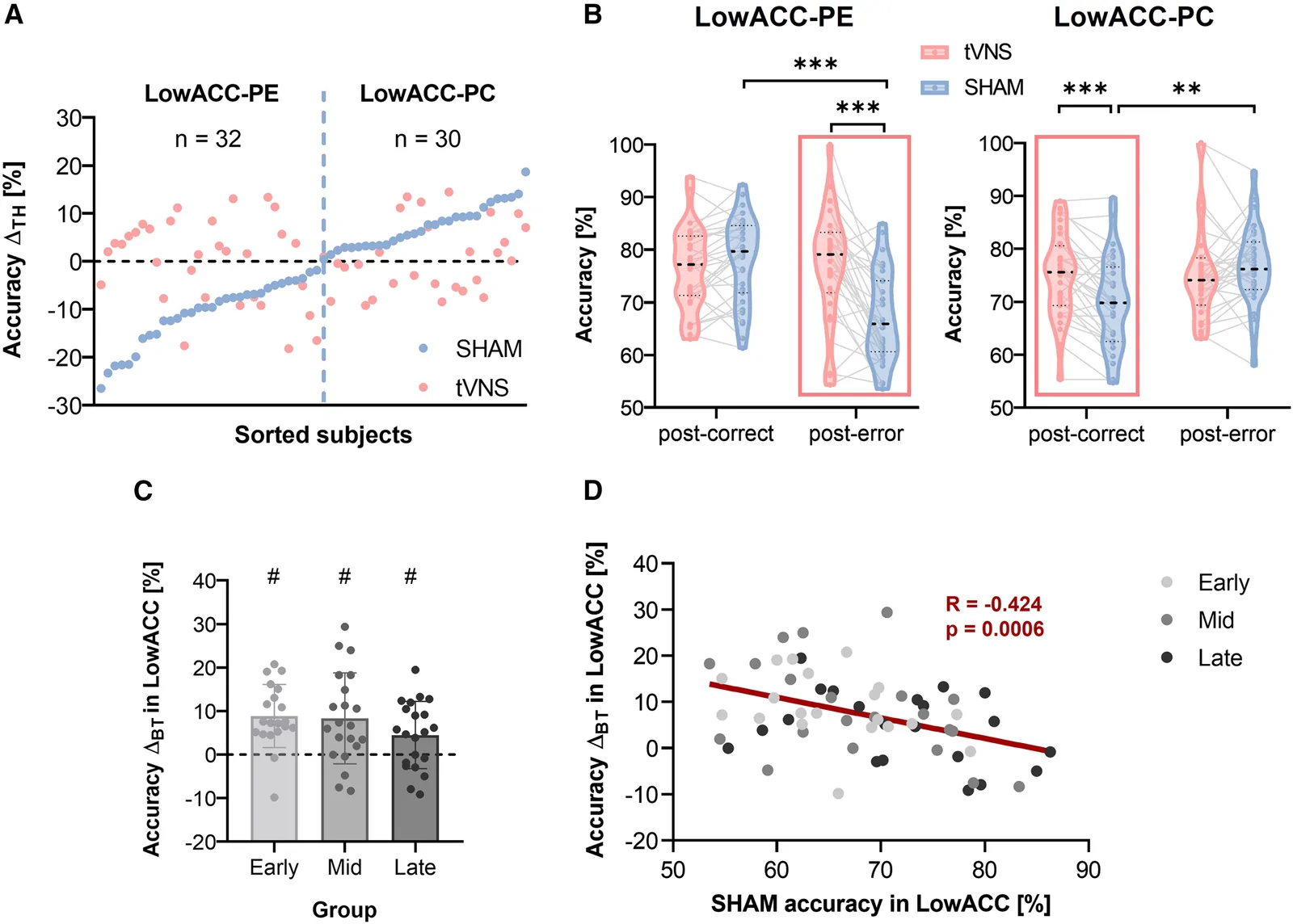
Accuracy as a function of trial-history (TH) in the RDM task (A) Definition of participant-cluster based on accuracy Δ_TH_ values. Dots represent Accuracy Δ_TH_ values in each participant, separated for tVNS (red) and SHAM (blue) blocks. Accuracy Δ_TH_ reflects the delta (Δ) of accuracy between post-error and post-correct trials (post-error minus post-correct). Participants were sorted and then clustered based on the sign of their Accuracy Δ_TH_ values in SHAM blocks. A negative value (left, *n* = 32) indicates lower accuracy in post-error trials (LowACC-PE cluster), while a positive value (right, *n* = 30) indicates lower accuracy in post-correct trials (LowACC-PC cluster). (B) Effect of tVNS on accuracy in each participant-cluster, as a function of trial-history. The accuracy data (in %) features median values as black dotted lines, distributions as violin plots, and individual data as thin gray lines, separated for tVNS (red) and SHAM (blue) blocks in each group. Note that tVNS selectively improved accuracy in post-error trials for LowACC-PE participants (marked with a red rectangle in the left image), while selectively improved accuracy in post-correct trials for LowACC-PC participants (marked with a red rectangle in the right image). (C) Accuracy Δ_BT_ in LowACC trials across groups. Accuracy Δ_BT_ represents the delta of accuracy between tVNS and SHAM blocks (tVNS minus SHAM). Data were specifically derived from LowACC trials (post-error trials in LowACC-PE participants and post-correct trials in LowACC-PC participants). Individual data in Group_Early_, Group_Mid_, and Group_Late_ are represented by light, medium, and dark gray dots, respectively. Bar plots indicate the Mean ± SEM. Note that Accuracy Δ_BT_ values were comparable across groups, and each group showed significant values above 0. (D) Correlation between SHAM accuracy and Accuracy Δ_BT_ in LowACC trials. Note that participants who exhibited the lower accuracy in SHAM blocks were those who benefited the most from the tVNS, as reflected by the greater Accuracy Δ_BT_ in these subjects. ∗∗: *p* < 0.01. ∗∗∗: *p* < 0.001. #: *p* < 0.05.

Following this finding, we restricted the next analyses to the trial type showing lower accuracy in SHAM blocks (LowACC trials) for each participant. That is, we selected the post-error trials in participants of the LowACC-PE cluster and the post-correct trials in participants of the LowACC-PC cluster and computed the Accuracy Δ_BT_, defined as the difference in accuracy between Block-Type (BT: tVNS−SHAM) for these LowACC trials. The value of Accuracy Δ_BT_ reflects the degree to which tVNS enhanced accuracy in each participant. We first compared these values among the three different groups (Early, Mid, Late), but the one-way ANOVA did not reveal any significant difference (F(2, 59) = 1.598, *p* = 0.211, see Figure 4C). In fact, *t* tests against zero showed that the Accuracy Δ_BT_ was significant in each group (all t > 2.664, all *p* < 0.045). Hence, tVNS rescued accuracy in LowACC trials, regardless of the timing at which it was applied. Finally, in the last analysis, we pooled all participants from the three groups together and ran Pearson’s partial correlation analysis to investigate the degree to which the Accuracy Δ_BT_ in LowACC trials depended on how low the accuracy was in these trials during SHAM blocks. Interestingly, participants who exhibited the lowest accuracy in SHAM blocks were those who benefited the most from the tVNS, as reflected by the greater Accuracy Δ_BT_ in these subjects (R = −0.424, *p* = 0.0006; Figure 4D). To account for potential regression-to-the-mean effects arising from the shared SHAM accuracy term, we additionally applied Oldham’s correction by correlating Accuracy ΔBT with the average accuracy across tVNS and SHAM conditions in LowACC trials. The relationship remained significant after correction (R = −0.351, *p* = 0.017). Hence, tVNS helped participants in low accuracy trials, especially in those showing the lowest accuracy, regardless of the timing at which it was applied.

The Block-Type × Trial-History × Accuracy Δ_TH_ interaction was not significant for RTs (F(1, 17423) = 0.294, *p* = 0.587), but the linear mixed-effects model revealed a significant Group × Trial-History interaction (F(2, 11482.7) = 5.582, *p* = 0.004). Overall, participants displayed longer RTs following errors compared to correct trials, consistent with the “post-error slowing” phenomenon repeatedly reported in past research.^32^ This post-error slowing effect reached significance in Group_Mid_ and Group_Late_ (Group_Mid_: t(48.8) = −3.954, *p*_*tukey*_ = 0.003; Group_Late_: t(43.6) = 5.341, *p*_*tukey*_ < 0.001; see Figure 5, middle and right images). In Group_Early_, although a similar trend was visually present (post-correct: 1.103 ± 0.059s, post-error: 1.144 ± 0.062s), the effect was not statistically significant (t(42.5) = −2.243, *p*_*tukey*_ = 0.24; see Figure 5, left image). Importantly, none of these effects interacted with the Block-Type (all F < 2.203, all *p* > 0.111), confirming the absence of a tVNS effect on RTs (see Figure 3B). In conclusion, tVNS selectively enhanced decision accuracy without incurring a cost on RT, as also reflected by the tVNS-related upward shift of the conditional accuracy curve when specifically considering LowACC trials in all experimental groups (see Figure 6A). We further analyzed the Accuracy-AUC Δ_BT_ (tVNS minus SHAM) derived from these conditional accuracy curves involving only LowACC trials. A one-way ANOVA revealed a significant group effect on Accuracy-AUC Δ_BT_ (F(2, 59) = 3.467, *p* = 0.038, Figure 6B), with the Group_Early_ exhibiting a significantly larger delta compared to the Group_Late_ (t = 2.601, *p*_*tukey*_ = 0.031). One-sample *t* tests against zero further confirmed that both Group_Early_ (t = 5.743, *p* < 0.001) and Group_Mid_ (t = 3.857, *p* < 0.001) exhibited significantly positive Accuracy-AUC Δ_BT_ values, while the effect in Group_Late_ did not reach significance (t = 1.895, *p* = 0.073). These results suggest that when only considering LowACC trials, the overall magnitude of tVNS-induced accuracy enhancement across the RT range was more pronounced when stimulation was applied earlier during the decision process.

**Figure 5:**
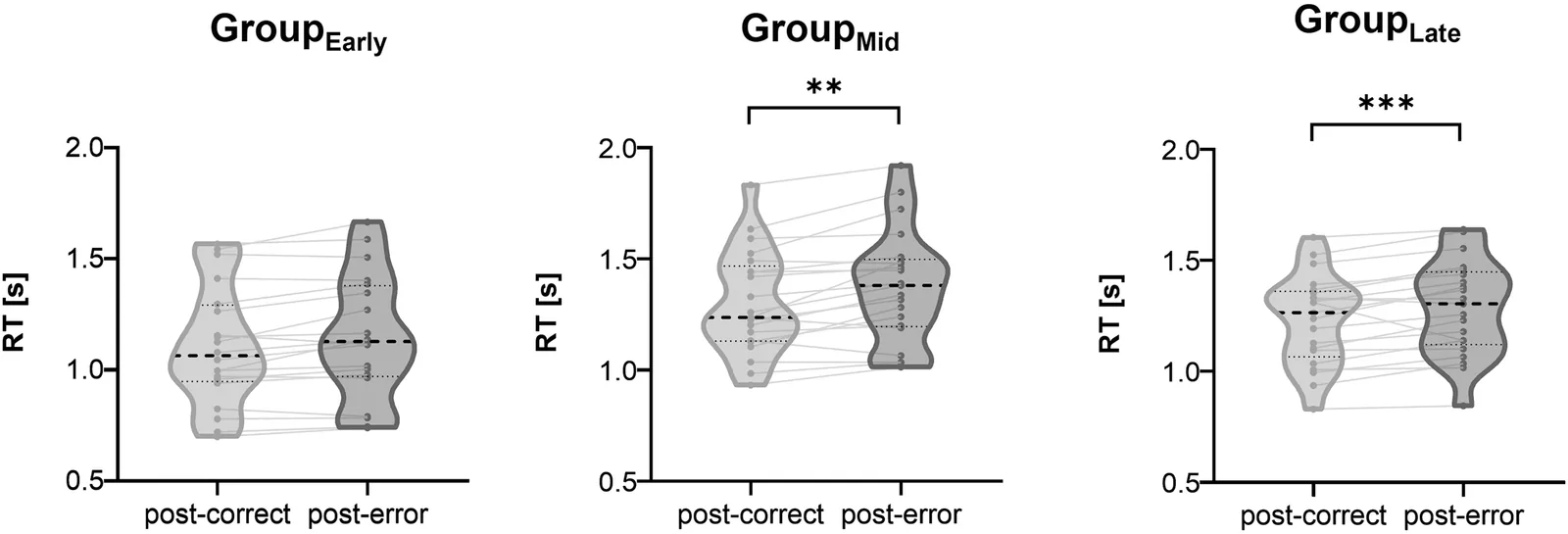
Reaction time (RT) as a function of Trial-History in the RDM task The RT data features median values as black dotted lines, distributions as violin plots, and individual data as thin gray lines. Note significantly longer RTs in post-error (dark gray) compared to post-correct (light gray) trials in Group_Mid_ (middle image) and Group_Late_ (right image), while participants in Group_Early_ tended to make slower responses without significance (left image). ∗∗: *p* < 0.01. ∗∗∗: *p* < 0.001.

**Figure 6:**
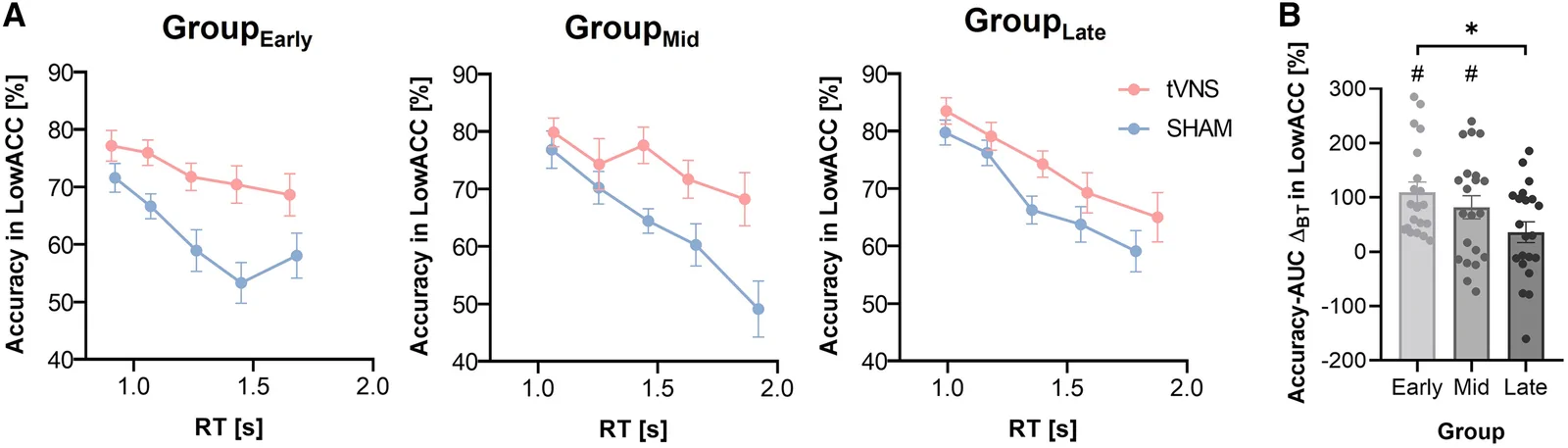
tVNS effect on accuracy across RT spectrum (A) Conditional accuracy curves in LowACC trials. Data are derived from LowACC trials in Group_Early_ (left image), Group_Mid_ (middle image), and Group_Late_ (right image), with separate curves for tVNS (red) and SHAM (blue) blocks. Each data point represents the Mean ± SEM of accuracy calculated within RT bins. Note that the conditional accuracy curve under tVNS shifts upward relative to SHAM in all groups. (B) Accuracy-AUC Δ_BT_ in LowACC trials across groups. Bars represent the Accuracy-AUC Δ_BT_ as Mean ± SEM, while dots are used to display individual data, with values corresponding to the delta of conditional accuracy curves (tVNS minus SHAM) in LowACC trials integrated across RT bins. Note that Accuracy-AUC Δ_BT_ values were significantly larger than 0 in Group_Early_ and Group_Mid_, and that Group_Early_ exhibited significantly larger Accuracy-AUC Δ_BT_ values compared to Group_Late_. ∗: *p* < 0.05. #: *p* < 0.05.

### tVNS improved drift rate in conditions with initially lower accuracy and drift rate, regardless of its application time

We exploited the drift diffusion model (DDM) to explore the computational mechanisms underlying the selective behavioral effects. The DDM provides three key parameters: drift rate (reflecting the efficiency of sensory evidence accumulation),^33^ boundary (representing decision-making criteria),^34^ and non-decision time (capturing processes other than decision-making, such as motor execution).^35^ Here, we used a refined DDM with linearly collapsing boundaries, where a lower boundary intercept can be interpreted as a higher initial urgency, and a steeper boundary slope can be seen as a stronger increase in urgency to respond as time progresses, leading to a less skewed RT distribution and a drop in accuracy over time.^34^ A two-way repeated measures ANOVA on drift rate, with Block-Type (tVNS, SHAM) and Trial-Type (LowACC, HighACC) as within-subject factors, revealed significant main effects (Block-Type: F(1, 48) = 7.733, *p* = 0.008; Trial-Type: F(1, 48) = 18.960, *p* < 0.001) and a Block-Type × Trial-Type interaction (F(1, 48) = 28.883, *p* < 0.001). Post-hoc comparisons showed that drift rate was generally lower in LowACC compared to HighACC trials for SHAM blocks (t = −6.454, *p*_*holm*_ < 0.001, see Figure 7A). Crucially, tVNS significantly increased drift rate relative to SHAM in LowACC trials (t = 5,436, *p*_*holm*_ < 0.001), effectively restoring it to levels comparable to HighACC trials in tVNS blocks (t = 1.590, *p*_*holm*_ = 0.237). In contrast, no significant difference in drift rate was observed between tVNS and SHAM in HighACC trials (t = −2.100, *p*_*holm*_ = 0.123), suggesting that the tVNS effect was specific to conditions in which baseline performance was suboptimal. For the remaining model parameters, including boundary intercept, boundary slope, and non-decision time, no significant tVNS effects were found (*p* > 0.204; see Figures 7B–7D). Detailed statistics for DDM parameters, including effect sizes and confidence intervals, are provided in Table S5. Although a Trial-Type × Block-Type interaction was observed for boundary slope, post hoc comparisons did not reveal significant tVNS-related differences within either LowACC or HighACC trials (Table S5).

**Figure 7:**
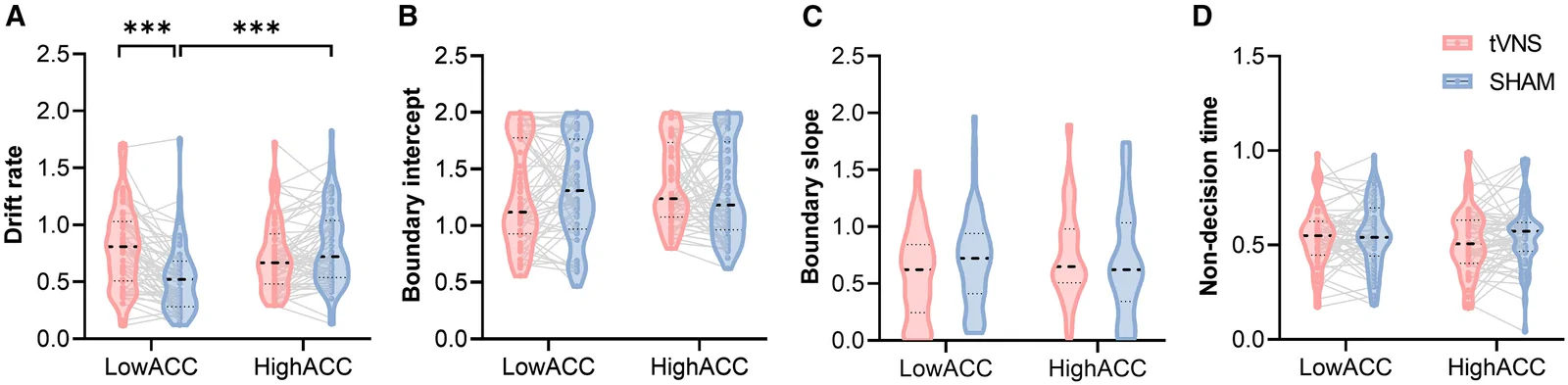
Drift diffusion model (DDM) parameters The DDM data features median values as black dotted lines, distributions as violin plots, and individual data as thin gray lines. (A) Drift rate. Note the lower drift rate in LowACC than in HighACC trials for SHAM blocks, and the selective tVNS increase in drift rate in the LowACC trials only. (B) Boundary intercept. Note the comparable values between tVNS and SHAM blocks in LowACC and HighACC trials. (C) Boundary slope. Same result as in B. (D) Non-decision time. Same result as in B. ∗∗∗: *p* < 0.001.

To further assess the robustness of the model-based findings, we fitted an additional model including a starting point bias parameter *z*∈[-1:1] (accumulation starts at *z*∗*a*_0_, with initial boundaries set to *a*_0_ and -*a*_0_). This starting point bias model yielded a slightly better fit to the data than the original model when fit per participant and block type (PEP = 1.00, BOR <0.001). Similar results were obtained when the data were further split according to trial history (post-correct trials: Protected Exceedance Probability, PEP = 0.57, Bayesian Omnibus Risk, BOR = 0.85; post-error trials: PEP = 0.70, BOR = 0.15). Importantly, the pattern of results remained unchanged, with tVNS effects selectively expressed in drift rate, while no significant main effects of Block-Type, Trial-Type, or Block-Type × Trial-Type interaction were observed for the starting point bias parameter (*p* > 0.178).

### tVNS induced a similar pupil dilation in trial types with low or high accuracy

Finally, we asked whether the selective effect of tVNS on LowACC trials could be accounted for by a greater tVNS-induced increase in pupil-linked arousal in these trials, compared to HighACC trials. To address this question, we separately examined stimulation-aligned Pupil-AUC Δ_BT_ (Figure 2D) and decision-aligned Pupil-AUC Δ_BT_ (Figure 2E) measures for the two trial types (LowACC, HighACC) in each participant. These data were then analyzed separately using repeated-measures ANOVAs with Trial-Type (LowACC, HighACC) and Group (Early, Mid, Late) as factors. Apart from a significant main effect of Group on the Stimulation-aligned Pupil-AUC Δ_BT_, as already described earlier (F(2, 58) = 6.901, *p* = 0.002; see Figure 8A), no significant main effects or interactions involving Trial-Type were detected in either analysis (all F < 1.252, all *p* > 0.268; see Figures 8A and 8B). We also looked at whether pupil baseline differed at the onset of these trial types, but this was not the case either: there was no main effect of Trial-Type (F(1, 20.7) = 0.149, *p* = 0.704) or Block-Type × Trial-Type interaction (F(1, 20.2) = 1.019, *p* = 0.325) on pupil baseline (see Figure S3C). Together, these results suggest that tVNS induced similar stimulation-aligned and decision-aligned pupil responses in trials with initially low or high accuracy, despite selectively enhancing decision accuracy only in the former condition. Thus, the selective tVNS-related enhancement in accuracy was not accompanied by a corresponding selective increase in pupil responses. Instead, tVNS seems to have triggered a consistent increase in arousal engagement across trial types.

**Figure 8:**
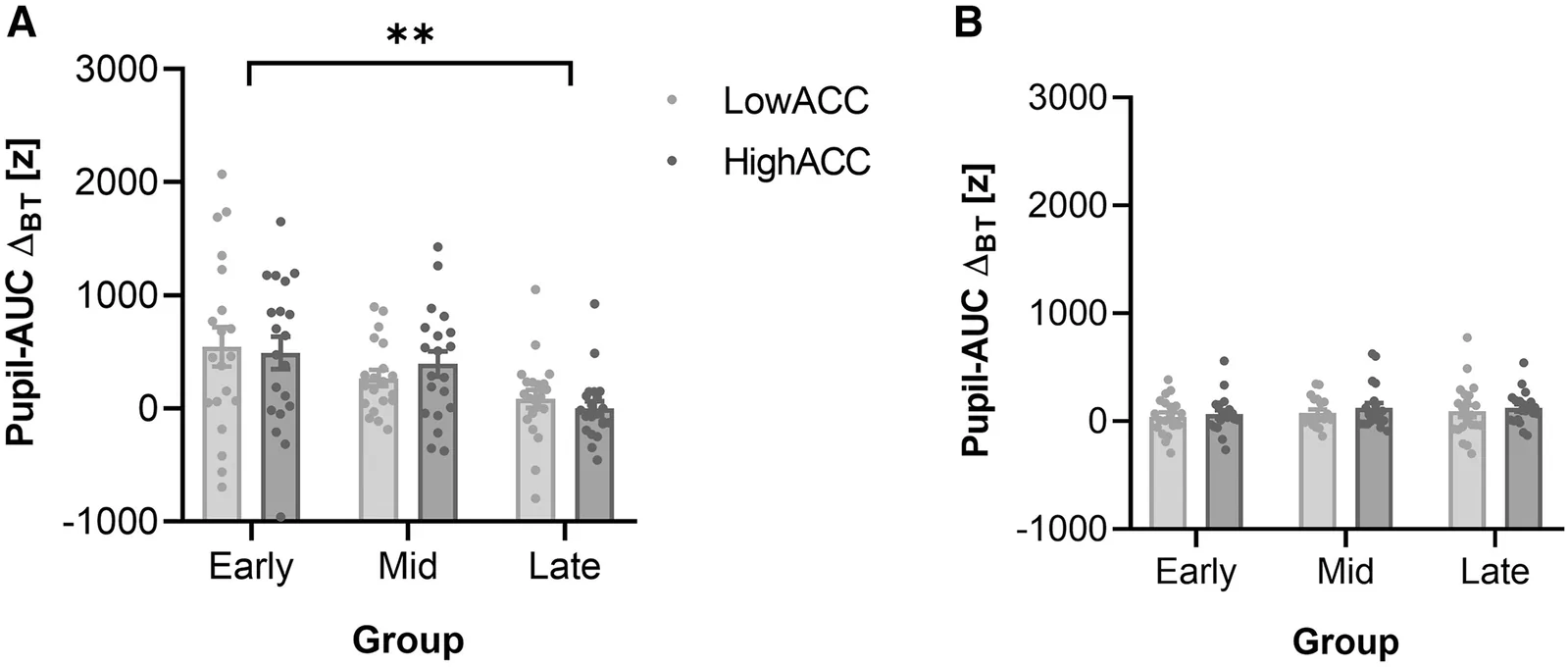
tVNS effect on pupil dilation across trial types and experimental groups These graphs show the overall effect of tVNS on pupil dilation, expressed as the Pupil-AUC Δ_BT_. Individual dots and bars represent values for LowACC trials (light gray; post-error trials in LowACC-PE participants and post-correct trials in LowACC-PC participants) and HighACC trials (dark gray; post-correct trials in LowACC-PE participants and post-error trials in LowACC-PC participants) across Group_Early_, Group_Mid_, and Group_Late_. Bar plots indicate mean ± SEM. (A) Pupil-AUC Δ_BT_ aligned to stimulation onset. While a significantly higher Pupil-AUC Δ_BT_ was observed in Group_Early_ when compared to Group_Late_, all groups showed comparable Pupil-AUC Δ_BT_ between LowACC and HighACC trials. (B) Pupil-AUC Δ_BT_ during the Decision phase. All groups showed comparable Pupil-AUC Δ_BT_ between LowACC and HighACC trials. ∗∗: *p* < 0.01.

## Discussion

This study investigated the behavioral impact and computational mechanisms of transiently enhancing arousal-related neuromodulatory activity using online tVNS, applied at three distinct time points across groups performing perceptual decisions in an RDM task. Across all groups, tVNS reliably increased pupil dilation relative to SHAM, indicating effective pupil-linked arousal engagement during the task. Behaviorally, tVNS consistently improved decision accuracy without affecting response speed, supporting a gain-related enhancement of evidence accumulation during decision-making rather than an urgency-driven acceleration of response commitment. Improvements were most prominent in trials characterized by initially low accuracy under SHAM, indicating that the behavioral benefit of tVNS emerged selectively under suboptimal performance states. DDM analyses further revealed that this selective accuracy enhancement is mediated by an increase in drift rate, reflecting more efficient evidence accumulation under tVNS. Notably, stimulation applied early in the trial elicited the most sustained pupil dilation, initiated during the Fixation phase and extending into the Decision period, resulting in a comparable magnitude of pupil response across groups during decision-making. Relatedly, while the tVNS effect on accuracy was quite consistent across groups, it was also most pronounced when tVNS was delivered early in the trial.

Building on our previous findings,^27^ the present study confirms that tVNS improves decision accuracy without compromising RTs, consistent with previous work suggesting that tVNS exerts gain-related effects on cognitive processing^20^ and with the broader gain hypothesis of arousal function.^1^^,^^36^^,^^37^ By applying tVNS at distinct phases of the decision process in a larger sample (*n* = 62), we provide strong evidence for the robustness of this effect and demonstrate its generalizability across different stimulation timings. Importantly, our results also indicate that the behavioral benefits of tVNS are not uniform across all trials but instead emerge selectively in trials associated with relatively lower accuracy under SHAM. Specifically, for participants who showed reduced accuracy following errors in SHAM blocks, tVNS selectively enhanced performance in post-error trials. Conversely, for those whose accuracy was lower after correct responses under SHAM, performance improvements by tVNS emerged primarily in post-correct trials. These patterns suggest that tVNS reinforced performance in contexts where endogenous control was weakest, although the mechanisms underlying these decrements likely differed across subgroups. When task accuracy was generally high, errors occurred infrequently and thus acted as salient and unexpected events that transiently disrupted attentional focus and cognitive control.^6^^,^^38^^,^^39^^,^^40^ Consistent with this interpretation, participants in our study who exhibited impaired post-error accuracy maintained relatively high accuracy in post-correct trials (mean = 79%), indicating that errors were rare and may have captured attention away from task-relevant information. In contrast, participants who showed poorer performance after correct responses had generally lower baseline accuracy (mean = 69%). This pattern mirrors findings from sustained attention tasks, where accuracy progressively declines across successive correct trials.^41^^,^^42^ Such declines have been attributed to attentional disengagement, likely driven by reduced performance monitoring or increased automatization during repetitive task execution.^43^^,^^44^^,^^45^ This mechanism likely underlies the reduced post-correction accuracy we observed in this participant cluster, where attention may have gradually faded in the absence of error-related feedback. Taken together, these findings suggest that tVNS selectively enhanced accuracy by reinforcing attentional control on task-relevant information during periods of weakened endogenous regulation, whether triggered by distraction following rare errors or by gradual disengagement in repetitive correct responding. These two performance states may reflect opposite deviations from optimal LC-NE activation, i.e., hyperactivation after errors and hypoactivation during prolonged task execution. This pattern is consistent with the classic inverted-U model of LC-NE function, whereby both excessive and insufficient noradrenergic activity impair cognitive control, and tVNS may restore intermediate, optimal levels that support goal-directed engagement.^1^^,^^4^^,^^29^

DDM analyses provide insight into the computational mechanisms underlying the behavioral effects of tVNS. This approach revealed a significant increase in drift rate under tVNS, while boundary parameters and non-decision time remained unaffected. Crucially, the effect on drift rate was confined to the low-accuracy trials, closely mirroring the selective accuracy improvements observed at the behavioral level. Specifically, in the SHAM condition, drift rates were lower in low-accuracy than high-accuracy contexts, indicating less efficient evidence accumulation during states of poorer performance.^46^ tVNS selectively restored drift rate in these low-accuracy trials, steepening the evidence accumulation to a level comparable to that in high-accuracy trials, thereby likely accounting for the observed improvement in decision accuracy. These drift-rate effects are consistent with enhanced evidence accumulation and provide computational support for previous gain-related accounts of tVNS,^20^ while the neurophysiological mechanisms underlying this gain-related processing remain to be established. Importantly, the present study did not directly measure LC activity or neural gain. Rather, pupil responses were used as an indirect index of arousal engagement, and gain-related processing was inferred from computational modeling of behavioral performance. Although a higher drift rate is often associated with both improved accuracy and faster responses, parameter-level effects may not necessarily translate into significant differences across all behavioral measures but depend on the overall dynamics of the decision process. Within the DDM framework, when model-derived evidence accumulation is initially inefficient, modest increases in drift rate may primarily improve the probability of reaching the correct boundary rather than markedly shortening decision time.^33^^,^^47^ Accordingly, the tVNS-induced enhancement in drift rate likely improved the quality of sensory evidence processing without altering the temporal dynamics of response execution. In contrast, if tVNS had acted on decision urgency, one would expect reduced decision boundaries or collapsing bounds, leading to faster but potentially less accurate responses.^16^^,^^48^ The absence of detectable changes in boundary parameters therefore provides little support for an urgency-driven modulation of decision processes. Nevertheless, recovery of the boundary slope parameter was weaker than that of the other model parameters, which may have limited sensitivity to detect subtle effects on this parameter. Future studies combining computational modeling with direct neural measures may help further dissociate gain and urgency-related mechanisms. Within the present dataset, however, the overall pattern of results was more consistent with a gain-related enhancement of model-derived evidence accumulation. Since the efficiency of evidence accumulation is tightly linked to attentional engagement,^49^^,^^50^ these findings suggest that pupil-linked arousal under tVNS may help stabilize attention when endogenous control is weakened, thereby facilitating the accumulation of task-relevant sensory evidence.

Interestingly, tVNS induced comparable pupil dilation in low- and high-accuracy contexts, even though its behavioral impact was confined to the former. In addition, tVNS-induced behavioral improvement was not systematically correlated with tVNS-induced pupil dilation, whether considering all trials or specifically LowACC trials. This suggests that the selectivity of the behavioral effect may not depend on the overall magnitude of pupil-linked arousal recruited by tVNS, but rather on how this arousal-related signal interacted with task-relevant cortical circuits, such as visual cortical areas, exerting stronger influences when these circuits were operating suboptimally. This interpretation is in line with recent evidence showing that auricular tVNS can alter directed cortical communication within task-relevant cortical networks during intentional actions, rather than exerting a uniform effect across the cortex.^51^ Finally, we did not observe any relationship between fluctuations in pupil dilation and decision behavior (RT and accuracy), even when considered across trials in sham blocks. Altogether, the absence of a clear relationship between pupil size and behavior makes it unlikely that our tVNS findings arose from a purely visual confound, namely that greater pupil diameter enhanced visual performance. This interpretation aligns with the view that arousal flexibly allocates cognitive resources according to internal demands.^28^^,^^31^^,^^52^^,^^53^ When attentional engagement is already high and neural processing is optimized in task-relevant processes, additional arousal engagement may offer little behavioral benefit and could even incur unnecessary metabolic costs.^54^^,^^55^^,^^56^ In contrast, under conditions of weakened endogenous control, such as attentional lapses or reduced task engagement, tVNS-induced arousal engagement may restore effective processing by stabilizing attention and facilitating the integration of task-relevant information. Thus, rather than producing a uniform effect on behavior, boosts of arousal seem to operate in a state-dependent manner, selectively enhancing performance when endogenous control mechanisms alone are insufficient to sustain optimal decision-making. It should be noted that, although participants were instructed to prioritize both speed and accuracy, only accuracy-related feedback was provided during the task. Therefore, the present design may have implicitly encouraged greater emphasis on accuracy, and future studies using feedback schemes that more strongly promote faster responding will be important for further evaluating the effects of tVNS in perceptual decision-making.

A notable aspect of our findings is the strong convergence of results across the three stimulation groups. Despite being applied at different time points corresponding to distinct phases of arousal engagement during the task, tVNS produced remarkably similar behavioral effects in all groups. These effects were characterized by improved decision accuracy without changes in RTs and by a shared pattern of selective enhancement in trials with lower SHAM performance. The consistency of these effects indicates that the behavioral efficacy of tVNS does not critically depend on the precise moment of stimulation, but rather likely on how stimulation interacts with the evolving cognitive dynamics of the task. Prior theoretical and empirical work has shown that the LC activity is modulated not only by external stimuli but also by ongoing task process and internal cognitive state.^5^^,^^57^^,^^58^^,^^59^ Consistent with this view, neurophysiological studies have demonstrated that the LC can sustain task-dependent modulation over extended timescales.^31^^,^^60^ Such context-sensitive modulation enables the arousal to influence behavior over sustained periods of engagement rather than being limited to transient stimulus-locked responses. Within this framework, the convergence of behavioral effects across groups suggests that the impact of tVNS is not determined by when stimulation begins, but by whether it coincides with periods of sustained task engagement, during which arousal engagement is actively recruited and temporally extended. Under such conditions, tVNS may amplify ongoing arousal dynamics that are already aligned with task structure, allowing its effects to persist across multiple phases of the decision process regardless of stimulation timing. While this interpretation is consistent with the observed pupil dynamics, future studies incorporating more direct measures of arousal-related neuromodulatory activity will be important for determining the extent to which these sustained effects reflect prolonged arousal engagement following tVNS.

Even if the behavioral effects of tVNS were broadly consistent across stimulation groups, participants in the Group_Early_ exhibited the greatest overall improvement in decision accuracy across the RT spectrum, as reflected by a greater upward shift in the conditional accuracy curve. This enhancement coincided with the most sustained pupil dilation, with Group_Early_ showing the highest overall pupil dilation aligned to stimulation onset. These findings suggest that delivering tVNS during the initial Fixation phase, when arousal engagement was still at baseline, may be particularly effective, potentially due to its alignment with the onset of task engagement. Prior research indicates that the efficacy of noradrenergic modulation depends on cognitive context, especially during periods when attention is being allocated, and control processes are being established.^61^^,^^62^^,^^63^ Applying stimulation at this preparatory stage, when internal control systems are configuring to support upcoming goal-directed behavior, may therefore provide a privileged window for modulating arousal-related neuromodulation. By enhancing arousal engagement early in the trial, tVNS may facilitate the establishment of a stable cognitive state and promote sustained attentional allocation throughout task performance. Such temporal alignment between early stimulation and task preparation could explain why neuromodulatory effects persisted throughout the decision process, ultimately leading to greater improvements in accuracy.

### Limitations of the study

Several limitations should be considered when interpreting the present findings. First, the study did not directly measure LC activity or neural gain. Pupil responses were used as an indirect index of arousal engagement, while gain-related processing was inferred from computational modeling of behavioral performance. Thus, future studies combining tVNS with more direct measures of neuromodulatory activity are needed to further establish the neurophysiological mechanisms underlying the observed effects. Second, recovery of the boundary slope parameter in the DDM was weaker than that of the other model parameters, which may have limited sensitivity to subtle urgency-related effects. Finally, although participants were instructed to prioritize both speed and accuracy, feedback was provided only for accuracy. This design may have implicitly encouraged greater emphasis on accuracy, and future studies using feedback schemes that more strongly promote faster responding may further clarify the effects of tVNS on the speed-accuracy tradeoff.

## Resource availability

### Lead contact

Further information and requests should be directed to the lead contact, Shiyong Su (contact: sushiyong@sjtu.edu.cn).

### Materials availability

This study did not generate new unique reagents.

### Data and code availability

• Behavioral data, drift diffusion model data, pupil data, and participant characteristics are publicly available on Zenodo at https://zenodo.org/records/21950273.
• This paper does not report original code.
• Supplementary materials are publicly available on Zenodo at https://zenodo.org/records/22750830.
• Any additional information required to reanalyze the data reported in this paper is available from the lead contact upon request.

## Acknowledgments

Shiyong Su was supported by grants from UCLouvain (FSR mobility grant: SW/AS/17846.2023) and from the 10.13039/501100002661Belgian National Fund for Scientific Research (FNRS: FC 48815). The research was also supported by an ARC grant from UCLouvain (Coaction) and research credits from FNRS (PDR UrgeToAct: T007023F and CDR-AROUSAT: J007424F).

## Author contributions

S.S.: conceptualization, methodology, software, validation, formal analysis, investigation, data curation, writing – original draft, writing – review and editing, visualization, project administration, and funding acquisition. T.V.: methodology, software, investigation, data curation. P.L.D.: methodology, software, and formal analysis. A.Z.: investigation, and writing – review and editing. J.D.: conceptualization, methodology, validation, resources, writing – review and editing, project administration, and funding acquisition.

## Declaration of interests

The authors declare no competing interests.

## STAR★Methods

### Key resources table

**Table:** 

REAGENT or RESOURCE	SOURCE	IDENTIFIER
**Deposited data**		

Behavioral data	This paper	https://zenodo.org/records/21950273
Fitted drift-diffusion model data	This paper	https://zenodo.org/records/21950273
Pupil data	This paper	https://zenodo.org/records/21950273
Subject information	This paper	https://zenodo.org/records/21950273
Supplementary materials	This paper	https://zenodo.org/records/22750830

**Software and algorithms**		

MATLAB R2022a	MathWorks	https://www.mathworks.com/products/matlab.html
JASP	JASP Team	https://jasp-stats.org/
Jamovi	The jamovi project	https://www.jamovi.org/
Psychophysics Toolbox	Psychtoolox.org	http://psychtoolbox.org/

### Experimental model and study participant details

#### Participants

Sixty-two healthy participants were considered in this study (37 women, 24.8 ± 0.78 years old). Data from 21 of these participants were already exploited in a recent study.^27^ All participants reported normal or corrected-to-normal vision, no history of neurological or psychiatric disorders, and no physical impairments. Before the experiment, they provided written informed consent and received financial compensation for their participation. They were all naive to the purpose of the study. The experimental protocol was approved by the Ethics Committee of the Université Catholique de Louvain (UCLouvain), Brussels, Belgium (approval numbers: 2018/22MAl/219 and 2023/13JUL/322), in accordance with the Declaration of Helsinki.

### Method details

#### Experimental setup

Experiments were conducted in a quiet and dimly lit room. The participants were seated at a table facing a 23-inch computer screen (60Hz refresh rate) positioned 60cm from their eyes, which displayed the visual stimuli of the random dot motion (RDM) task (see Figure 1A). They rested their forearms on the table, palms facing down, with the response keys F12 and F5 positioned under their left and right index fingers, respectively. During the task, tVNS/SHAM stimulation was delivered and pupillometry was continuously recorded, participants were instructed to minimize any movements and eye blinks during the recordings.

#### Transcutaneous vagus nerve stimulation

Pulses were generated using a Digitimer (Digitimer Ltd., model DS3), triggered by a Master8 device (A.M.P.I., model Master-8). As shown in Figure 1B, in the tVNS condition, the electrodes were placed at the left cymba conchae (anode) and tragus (cathode), which are heavily innervated by the auricular branch of the vagus nerve.^64^^,^^65^^,^^66^ In the SHAM condition, the electrodes were placed at the left earlobe, which is not expected to induce brainstem or cortical activation.^67^^,^^68^ Pulses (width, 200*μ*s) were delivered at a rate of 25Hz for 4s. To ensure similar sensory perception between tVNS and SHAM conditions, stimulation intensity was calibrated separately for each subject and condition, as also reported in.^27^ Specifically, participants underwent a “method of limits” procedure to determine the tVNS intensity at a level experienced as just below painful.^19^^,^^69^ Stimulation began at 0.1 mA and increased in 0.2 mA steps until participants reported a sensation of 9 on a 10-point scale (0 = no sensation, 10 = painful). This intensity was repeated before a decreasing series, where stimulation was lowered in 0.2 mA steps until the sensation dropped to 6 or below. The final tVNS intensity was set as the average of the intensities rated as 8. For SHAM intensity, the same procedure was used, but participants described the sensation relative to tVNS without providing numerical ratings. The final SHAM intensity was set as the average of the intensities they perceived as similar to tVNS at 8. Participants were monitored throughout the experiment to ensure that stimulation remained comfortable and well tolerated. No participant reported discomfort requiring interruption of the experiment or adjustment of the stimulation protocol. Detailed stimulation intensities for the tVNS and SHAM conditions are provided in Table S6.

#### Random dot motion task

Here, we aimed to test whether tVNS modulates decision-making via “gain” or “urgency” mechanisms, and whether its behavioral effects depend on stimulation timing relative to LC–NE engagement during the task. We used an RDM task implemented in Matlab (The Mathworks Inc. Natick, Massachusetts, USA) with the Psychophysics toolbox.^70^^,^^71^ As shown in Figure 1A, each trial began with a Fixation phase (6s in Group_Early_, 5s in Group_Mid_ and Group_Late_), during which participants fixated a central cross surrounded by stationary dots. This was followed by a Pre-decision phase, where dots moved randomly at 5°/s for 2.5-3.5s. Then, during the Decision phase, the fixation cross changed to “×” and a proportion of dots began moving coherently leftward or rightward. Participants were required to discriminate the coherent direction and provide their response by pressing the response key (left: F12, right: F5) with the left or right index finger within 2.5s. Participants were explicitly instructed to respond as fast and accurately as possible, concentrating on both speed and accuracy throughout the task. Task difficulty was determined by the proportion of coherently moving dots (coherence c’), which was individually adjusted for each participant (see below). After responding, dots returned to random motion during a Post-decision phase (1.5s), followed by a Feedback phase for 1.5s (arrow indicating correct direction; “missed!” if no response). This feedback was provided to facilitate performance monitoring and maintain task engagement throughout the experiment. Finally, an inter-trial interval (2s in Group_Early_ and 3s in Group_Mid_ and Group_Late_) consisting of a black screen was presented between trials. A 4-second train of tVNS or SHAM stimulation was applied in each trial, with participants randomly assigned to one of three groups based on the timing of stimulation within the trial. Stimulation began 2s, 3.5s, and 6s after trial onset for Group_Early_, Group_Mid_, and Group_Late_, respectively. Consequently, Group_Early_ (*n* = 20, 14 women; 23.4 ± 0.60 years old) received stimulation exclusively during the Fixation phase, Group_Mid_ (*n* = 21, 12 women; 25.7 ± 0.99 years old) during both the Fixation and Pre-decision phase, and Group_Late_ (*n* = 21, 11 women; 25.4 ± 0.75 years old) during the Pre-decision and Decision phases. Participants in each group received tVNS and SHAM stimulation in alternating blocks, allowing for within-subject comparisons. The data from Group_Mid_ were previously exploited in an earlier study.^27^ Note that the main task events were equivalent across groups except for minor timing adjustments in the Inter-trial and Fixation periods. As such, the Fixation phase was extended by 1s in Group_Early_ (6s instead of 5s), to allow the 4-s stimulation train to occur entirely within the Fixation phase while preserving a stimulation-free pupil baseline window. To maintain equivalent overall trial duration across groups, the inter-trial interval was shortened by 1 s in Group_Early_ (2 s instead of 3 s in the two other groups).

#### Experimental design

All participants came to the lab for a session of about 3 hours, which always started with some initial practice and difficulty calibration routines. The practice consisted of 4 blocks of 40 trials, with coherence levels (c’) progressively decreasing from 0.8 to 0.1 (0.8, 0.4, 0.2, 0.1) to familiarize participants with the task. Then, to account for individual differences in visual perception, the coherence level (c’) of moving dots in the RDM task was adjusted at the beginning of the experiment for each participant to achieve approximately 70% baseline accuracy. This was done in two calibration blocks of 100 trials, where coherence levels (c’ = 0.1, 0.2, 0.4, 0.6, 0.8) were presented in equal proportions and randomized across trials. A proportional rate diffusion model was then fitted to the data from these calibration blocks to estimate each participant’s psychometric function,^72^ from which the c’ value yielding 70% accuracy was interpolated and used in the main experiment. Following difficulty calibration, participants underwent a stimulation intensity calibration, where individual tVNS and SHAM intensities were determined to ensure consistent sensory perception across conditions (as described above). Participants were informed that stimulation could be delivered at different ear locations but were not informed of the active and sham stimulation conditions or the specific study hypotheses. During the main experiment, participants completed the RDM task in 8 blocks of 40 trials, with 4 blocks of tVNS and 4 blocks of SHAM stimulation presented in an alternating sequence. To control order effects, the sequence of the first 4 blocks was reversed in the second half of the session, and block order was counterbalanced across participants. Short breaks were provided between blocks to minimize fatigue.

### Quantification and statistical analysis

#### Behavioral measures and analyses

RT and accuracy in the RDM task were assessed as primary behavioral measures. Trials with missing responses or excessively fast reactions (< 450 ms) were excluded from both behavioral and pupillometric analyses. RT was defined as the time between the onset of coherent motion and the participant's key press, while accuracy was calculated as the percentage of correct responses across valid trials. On average, 142.3 ± 3.38 trials in the tVNS condition and 141.9 ± 3.41 trials in the SHAM condition were available for behavioral analyses across all participants.

Trials were categorized by Block-Type (BT: tVNS, SHAM) and were further classified based on whether they followed a correct choice or an error in the preceding trial (Trial-History, TH: post-correct, post-error). Moreover, we computed “Accuracy Δ_TH_”, reflecting each participant’s overall difference in accuracy (delta, Δ) between Trial-History (post-error minus post-correct) in SHAM blocks (see Figure 4A, blue dots). The Accuracy Δ_TH_ value was used as a covariate in our analyses; it also allowed us to ultimately categorize the participants in two clusters depending on whether they initially displayed a negative value, hence lower accuracy (LowACC) in post-error (PE) trials (*n* = 32, LowACC-PE) or a positive value, hence lower accuracy in post-correct (PC) trials (*n* = 30, LowACC-PC).

Statistical analyses of behavioral data were conducted in Jamovi (version 2.5.3) using generalized (Accuracy) and linear (RT) mixed-effects models. For accuracy analyses, generalized mixed-effects models were fitted using a binomial distribution with a logit link function, whereas RT was analyzed using linear mixed-effects models. The general model structure was:

Accuracy or RT ∼ Block-Type × Group × Trial-History × Accuracy Δ_TH_ + (1|Participant)

Where Block-Type (tVNS, SHAM), Group (Early, Mid, Late), and Trial-History (post-correct, post-error) were included as fixed factors, Accuracy Δ_TH_ was included as a covariate, and Participant was included as a random intercept. Participant-Cluster (LowACC-PE, LowACC-PC) was incorporated when necessary. Follow-up ANOVAs (and t-tests when necessary) were also run on Accuracy Δ_BT_ measures computed in each participant, defined as the difference in accuracy between Block-Type (tVNS minus SHAM). Moreover, relationships between variables were tested using Pearson’s partial correlation analyses.

To capture how tVNS influenced accuracy as a function of RT, we computed conditional accuracy curves. These conditional accuracy curves were generated separately for tVNS and SHAM blocks using a sliding-window approach. That is, for each participant, trials were sorted by RT within each block type, and mean accuracy was computed within overlapping windows spanning 40% of the RT range, shifting in 15% increments. This procedure yielded a total of 5 bins per participant and condition. The resulting conditional accuracy curves for tVNS and SHAM blocks were first computed at the individual level and then averaged across participants. SEM was calculated on accuracy values across participants for each bin. To quantify the overall effect of tVNS on these curves, we calculated the area under the accuracy curve (AUC) between Block-Type (tVNS minus SHAM) using trapezoidal integration over RT bin centers averaged across tVNS and SHAM conditions, referred to as Accuracy-AUC Δ_BT_. Accuracy-AUC Δ_BT_ values were compared across groups using a one-way ANOVA. Results are expressed as means ± SEM throughout the manuscript, except for violin plots where medians and interquartile ranges (IQR) are shown. To control multiple comparisons, Tukey-adjusted post hoc tests were used following significant mixed-effects model effects as well as ANOVAs, whereas Bonferroni corrections were used for t-tests when appropriate. An alpha level of 0.05 was used for all statistical tests.

#### Drift diffusion modeling

The DDM was fitted to the RT and Accuracy data using a variant that includes linearly collapsing decision boundaries. The decision boundary during each trial was modeled as:

$$a_{t}=a_{0}-ut$$

Where *a*_*t*_ is the boundary height at time *t*, *a*_0_ is the initial decision boundary (further referred to as boundary intercept), and *u*≥0 is the collapse rate of the boundary (further referred to as boundary slope). The inclusion of collapsing boundaries was justified by the response deadline (2.5s) imposed on participants.^73^ Sanitary model comparison revealed that collapsing boundaries yielded a better fit to the data compared to fixed boundaries (mean ΔBIC, Bayesian Information Criterion, over participant and block type in favor of the collapsing boundary model: 6.94, protected exceedance probability of the collapsing boundary model = 1.00). The drift rate, boundary intercept, boundary slope, and non-decision time were estimated separately for each participant within each condition defined by Block-Type (tVNS, SHAM) and Trial-History (post-correct, post-error). Quantile optimization was used for fitting the DDM.^74^ The optimization was performed by a differential evolution algorithm as implemented in the DEoptim package in R.^75^ The population size was set at ten times the number of free parameters. Convergence was met if no improvement in the objective function was found in the last 100 generations. To ensure a reliable interpretation of estimated parameters, a parameter recovery analysis was performed. We simulated the data of 1000 simulated participants using parameter values randomly sampled from their typical range. We managed to recover the parameters with as few as 25 trials: all correlation coefficients between generated and estimated parameters were larger than 0.73, except for the boundary slope (r = 0.53). As a result, we excluded 10 subjects with fewer than 25 post-error trials, leaving 52 subjects with an average of 37.4 ± 1.41 and 40.1 ± 1.57 post-error trials in the tVNS and SHAM conditions, respectively. For post-correct trials, all 62 subjects were included, with an average of 107.9 ± 3.03 trials in the tVNS condition and 105.2 ± 3.07 trials in the SHAM condition. Two-way repeated-measures ANOVAs were conducted to compare estimated DDM parameters across Block-Type (tVNS, SHAM), and Trial-Type (LowACC trials: post-error trials in the LowACC-PE cluster and post-correct trials in the LowACC-PC cluster, HighACC trials: post-correct trials in the LowACC-PE cluster and post-error trials in the LowACC-PC cluster). Holm-adjusted post hoc comparisons were applied following repeated-measures ANOVAs.

#### Pupillometry data acquisition and analyses

Pupil size was recorded binocularly at 1000 Hz using an Eyelink 1000+ eye-tracker (SR Research) and analyzed in MATLAB. Blink periods were identified and removed by linearly interpolating values 100ms before and after each blink, as implemented in custom MATLAB scripts. The pupil signal was then low-pass filtered using a 10 Hz fourth-order Butterworth filter with zero-phase shift. To ensure data quality, trials where interpolated data exceeded 50% of the total samples were excluded. For subsequent analyses, the eye with the more stable pupil signal, determined by the lower proportion of interpolated data and smaller deviation, was selected for each participant, and the same eye was used across all experimental conditions. Blink rate (tVNS: 5.63 ± 0.34%; SHAM: 5.87 ± 0.41%) and interpolation rate (tVNS: 3.49 ± 0.29%; SHAM: 3.15 ± 0.45%) were comparable across stimulation conditions (*p* > 0.34). After these pre-processing procedures, one participant in Group_Mid_ was excluded (remaining *n* = 20 in Group_Mid_ for pupil analyses). Additionally, after excluding trials with no response or very fast responses, an average of 140.7 ± 3.49 in tVNS blocks and 139.7 ± 3.51 trials in SHAM blocks were included for pupil analyses, with no significant difference in trial sample size between the two conditions (*p* = 0.54). To avoid arbitrary units, we converted pupil size into z scores computed across all conditions within each subject. Pupil baseline was defined as the average pupil size from 1-2s after trial onset in the three groups (see Figures 2A–2C, left panel). Then, to assess how tVNS influenced arousal-related neuromodulatory activity during the RDM task, dynamics of pupil dilation in tVNS and SHAM blocks were analyzed in the three groups by considering group-specific 11-second windows aligned to stimulation onset: [-1 10]s for Group_Early_, [-3 8]s for Group_Mid_, and [-5 6]s for Group_Late_ (see Figures 2A–2C, middle panel). Moreover, to specifically consider pupil dilation during decision-making in all groups, we also used a 5-second window aligned to coherent dot motion onset ([-1 4]s in all groups, see Figures 2A–2C, right panel).

For the statistical analysis of pupil baseline, we ran a linear mixed-effects model with the following structure:

Pupil baseline ∼ Block-Type × Trial-History + (1|Participant)

where Block-Type (tVNS, SHAM) and Trial-History (post-correct, post-error) were included as within-subject factors, and Participant was included as a random intercept. Since pupil size was normalized using z-scores, the absolute z value of pupil baseline is not meaningful, and Group was therefore not included as a factor in this analysis. Then, for pupil dilation, we first used paired t-tests (Block-Type: tVNS, SHAM) with cluster-based Monte-Carlo permutations (1000 iterations), identifying periods of significant differences between tVNS and SHAM blocks for each group. This was done both on the 11-second window aligned to stimulation onset and on the 5-second window aligned to coherent dot motion onset. After that, to compare the overall impact of tVNS on pupil dilation depending on its application time during the task, we computed the area under the curve (AUC) between the tVNS and SHAM pupil response traces (Pupil-AUC Δ_BT_) aligned to stimulation onset, over the whole period of significant differences in the three groups. We also computed the Pupil-AUC Δ_BT_ aligned to coherent dot motion onset. In this case, the analysis window for each participant spanned from the coherent dot motion onset to the participant's average RT to assess group differences in the tVNS effect during the Decision phase. These two pupil response traces measures were analyzed using separate repeated measures ANOVAs with Group (Early, Mid, Late) as a between-subject factor, Trial-History (post correct, post-error) as a within-subject factor, and Accuracy Δ_TH_ in SHAM blocks included as a covariate, consistent with the approach used in the behavior analysis.

To assess whether pupil responses varied across different performance contexts, we performed complementary analyses on several pupil-related measures. For the two Pupil-AUC Δ_BT_ measures (aligned to stimulation onset and to coherent dot motion onset), repeated-measures ANOVAs were conducted with Trial-Type (LowACC, HighACC) and Group (Early, Mid, Late) as factors. For pupil baseline, a linear mixed-effects model was run with Block-Type (tVNS, SHAM) and Trial-Type (LowACC, HighACC) as within-subject factors.

Finally, in a control analysis, we also checked whether behavior (accuracy, RT) naturally varies as a function of pupil dilation during the decision phase. To do so, we considered data in SHAM blocks and used generalized (accuracy) or linear (RT) mixed-effects models with the following structure:

Accuracy or RT ∼ Group × Pupil dilation (during decision-making) + (1|Participant)

Where Group (Early, Mid, Late) was included as a fixed factor, Pupil dilation during decision-making as a covariate, and Participant was included as a random intercept. Pupil dilation during decision-making was further divided into four bins for post hoc analyses.

For continuous time-series pupil analyses, family-wise error rates were controlled using cluster-based permutation testing. When significant effects or interactions were observed, follow-up comparisons were conducted using Tukey-adjusted post hoc tests.
