# Supplementary material for: Transcutaneous vagus nerve stimulation boosts evidence accumulation during perceptual decision making
Source: iScience. 2026 Oct 8;29(11):117748. doi: 10.1016/j.isci.2026.117748 (PMC13676923; doi:10.1016/j.isci.2026.117748)
Supplement: Supplementary file 1 [file mmc1.pdf]

iScience, Volume *29*

Supplemental information

Transcutaneous vagus nerve stimulation boosts evidence accumulation during perceptual decision making

Shiyong Su, Thomas Vanvoorden, Pierre Le Denmat, Alexandre Zénon, and Julie Duque

Supplementary Materials

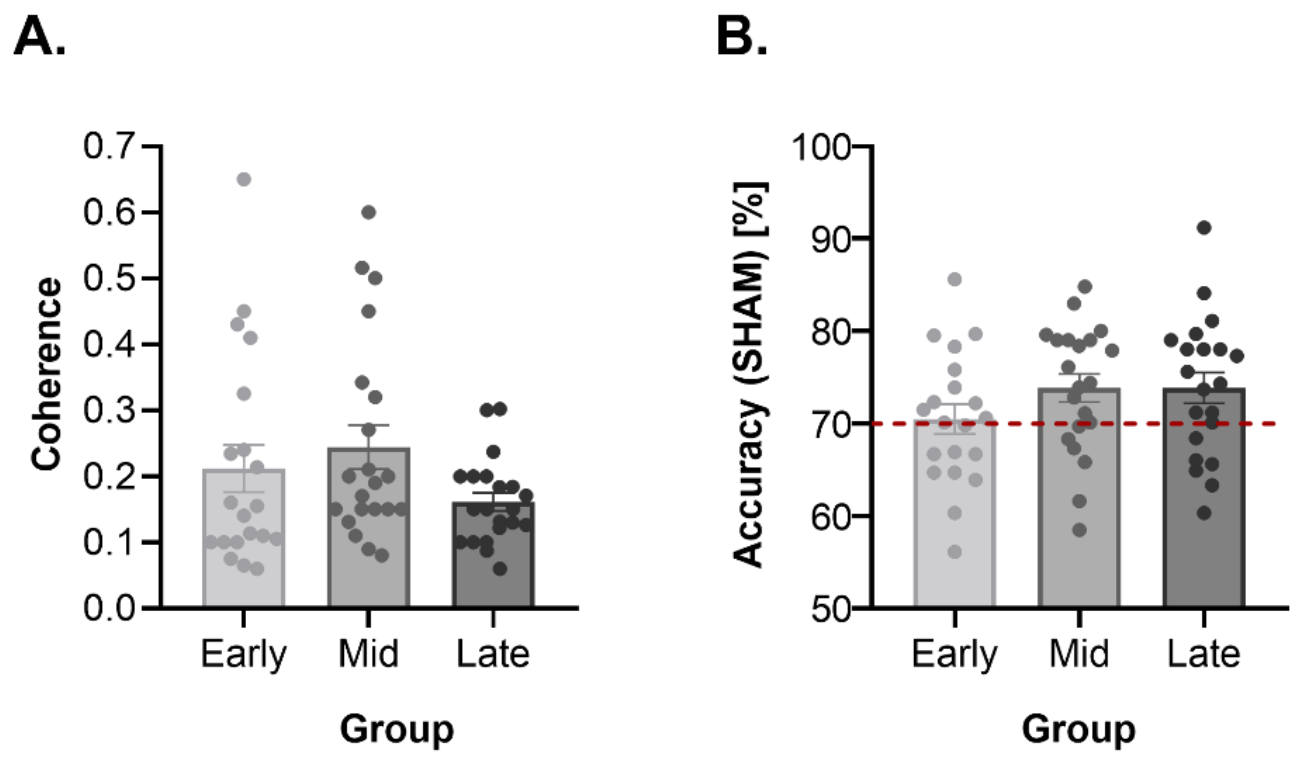

Supplementary Figure 1. Experimental calibration. Individual data in $Group_{Early}$, $Group_{Mid}$, and $Group_{Late}$ are represented by light, medium, and dark grey dots, respectively. Bar plots indicate the Mean ± SEM. **A. Coherence in the RDM task.** Note comparable coherence values in the three groups. **B. Decision accuracy in SHAM blocks.** Note the comparable decision accuracy of approximately 70% in SHAM blocks with calibrated coherence for the three groups.

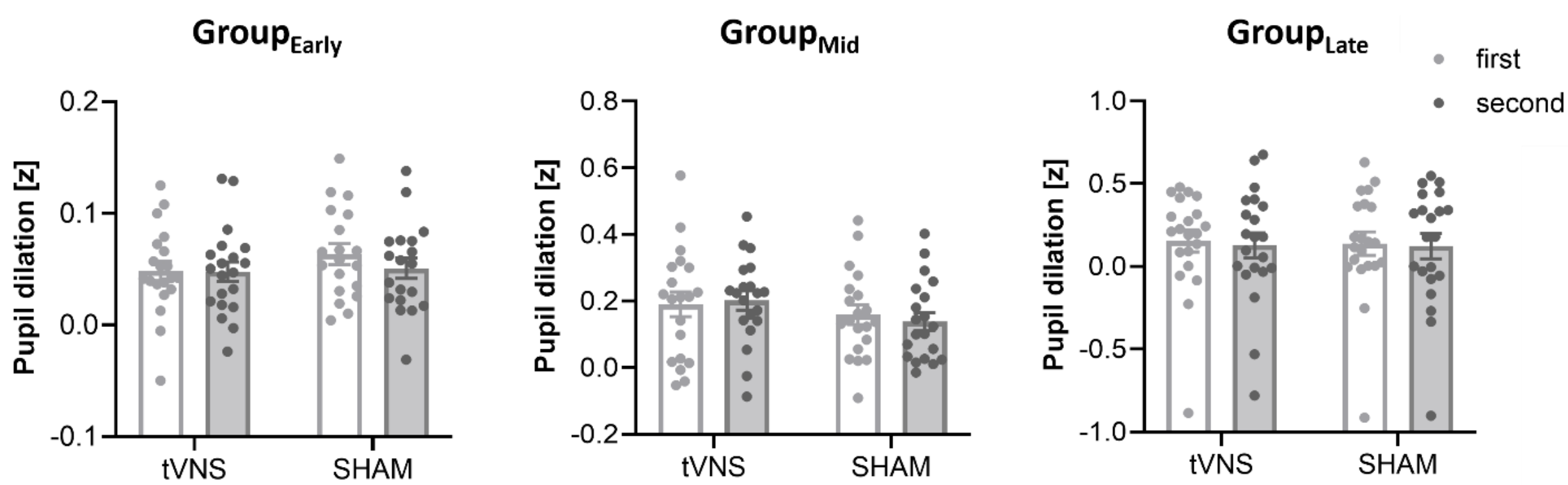

Supplementary Figure 2. Pupil dilation. Mean pupil dilation during the 500 ms preceding stimulation onset ([-0.5, 0] s) is shown separately for the first (light gray) and second (dark gray) halves of the experiment in $Group_{Early}$ (left panel), $Group_{Mid}$ (middle panel), and $Group_{Late}$ (right panel), under tVNS and SHAM conditions. The graphs display individual data as dots and bars indicate Mean ± SEM. Note that the pre-stimulation pupil increase remained comparable between the first and second half of the experiment across groups.

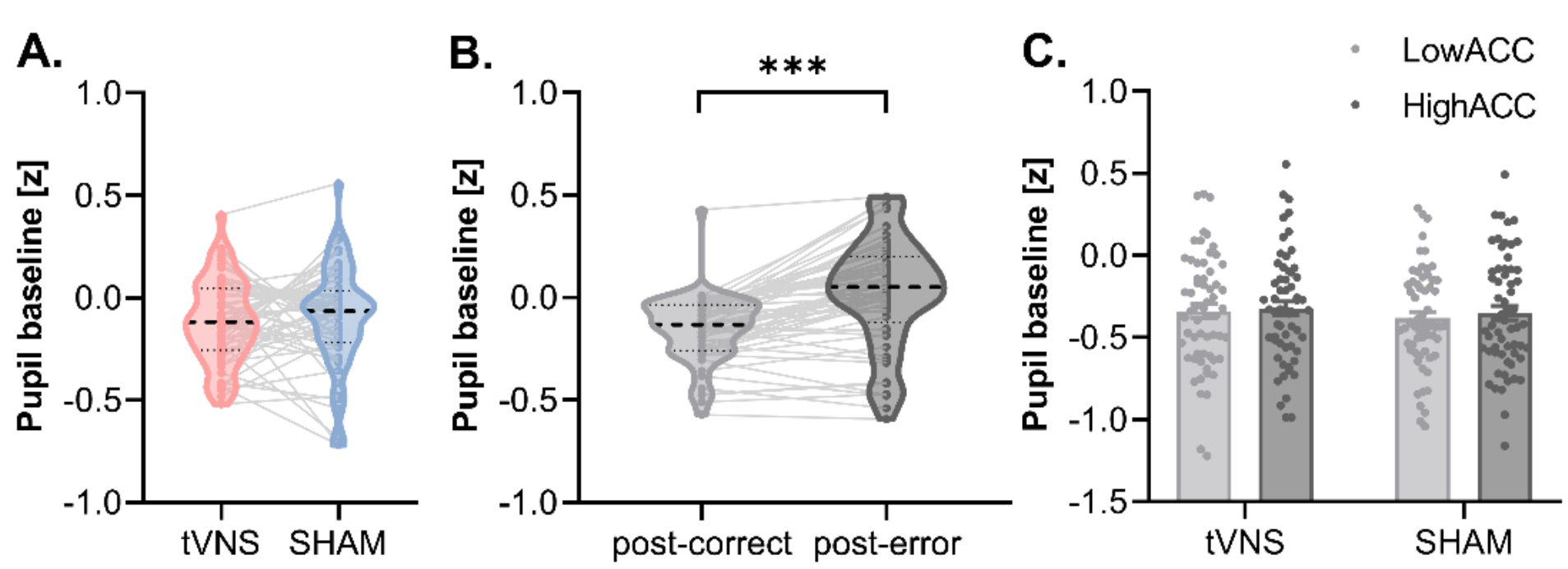

Supplementary Figure 3. Pupil baseline. The graphs display median values as black dotted lines, distributions as violin plots, and individual data as thin gray lines. **A. Block-Type effect on pupil baseline.** Note the comparable pupil baseline in tVNS (red) and SHAM (blue) blocks. **B. Trial-History effect on pupil baseline.** Note the greater pupil baseline in post-error (dark gray) than post-correct trials (light gray). **C. Trial-Type effect on pupil baseline.** Note the comparable pupil baseline in LowACC (light gray) and HighACC (dark gray) trials. ***: $p < 0.001$.

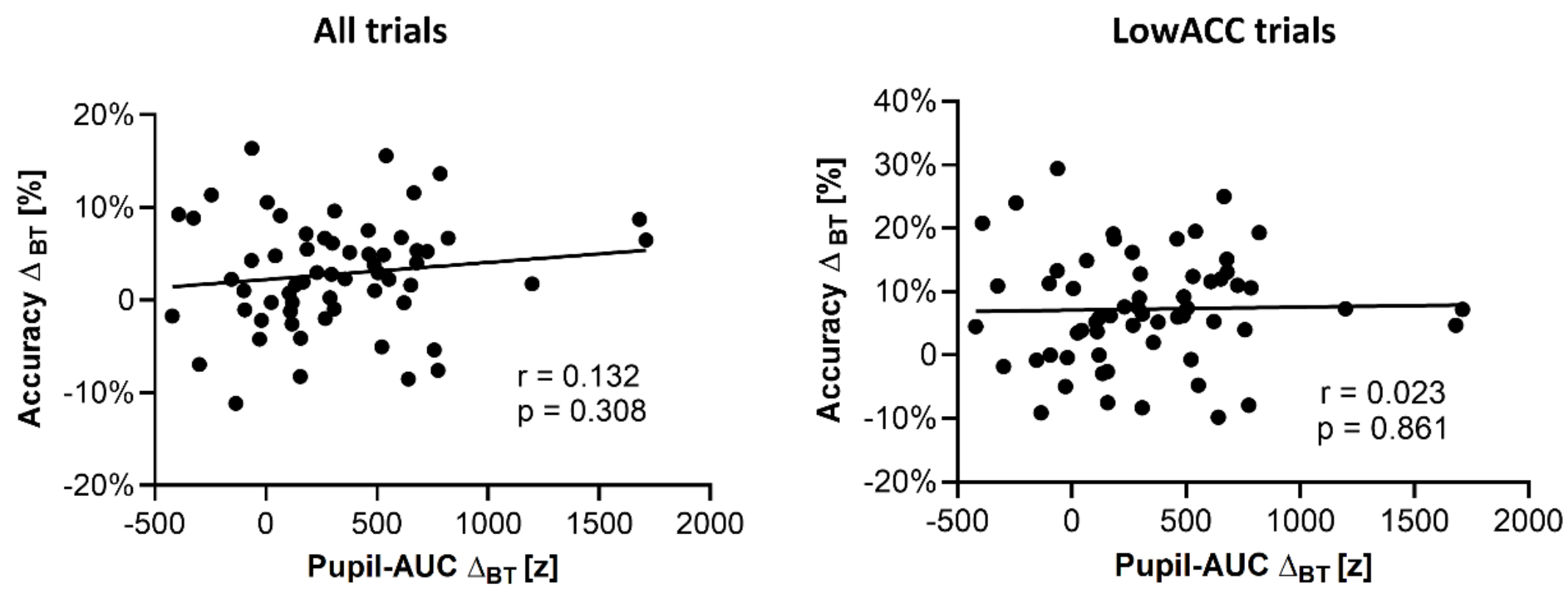

Supplementary Figure 4 Relationship between tVNS-induced pupil dilation and behavioral improvement. Correlation between the tVNS-induced change in pupil response (Pupil-AUC Δ_{BT}) and the tVNS-induced change in accuracy (Accuracy Δ_{BT}) across all trials (left panel) and LowACC trials (right panel). No significant relationship was observed in either analysis (all trials: r = 0.132, p = 0.308; LowACC trials: r = 0.023, p = 0.861).

Supplementary Table 1. Coherence calibration and baseline accuracy across groups.

Measure	$Group_{Early}$	$Group_{Mid}$	$Group_{Late}$	ANOVA F	p
Coherence (c')	0.218 ± 0.036	0.244 ± 0.033	0.161 ± 0.014	F(2,59) = 2.090	0.133
Accuracy (SHAM, %)	70.47 ± 1.59	73.82 ± 1.51	73.86 ± 1.65	F(2,59) = 2.681	0.077

Supplementary Table 2. Summary of pupil dilation dynamics under tVNS and SHAM conditions across groups.

Group	Blocks	Peak dilation (z)	Aligned to stimulation onset		Aligned to coherent dot motion onset	
			Peak time (s)	Return to baseline (s)	Peak time (s)	Return to baseline (s)
GroupEarly	tVNS	0.605	7.610	10.634	0.453	3.299
	SHAM	0.570	7.550	10.293	0.499	3.110
GroupMid	tVNS	0.658	5.132	7.878	0.597	3.257
	SHAM	0.593	5.100	7.838	0.572	3.264
GroupLate	tVNS	0.724	2.591	6.108	0.551	3.616
	SHAM	0.644	2.520	5.455	0.573	3.283

Supplementary Table 3. Main statistical effects on pupil responses.

		Effect	F	p	Effect size (ω^2)
**Pupil-AUC Δ_{BT}** (aligned to stimulation)	Repeated measures ANOVA	Group	6.901	0.002	0.063
		Trial-Type	0.002	0.967	0.000
		Group × Trial-Type	0.676	0.512	0.000
		**Comparison**	**Effect size (Cohen's)**	**95% CI**	**p**
	Post-hoc analyses	Early vs. Mid	0.363	[-0.270 0.996]	0.334
		Early vs. Late	0.925	[0.270 1.580]	0.001
		Mid vs. Late	0.562	[-0.071 1.195]	0.074
		**Effect**	**Effect size (Estimate β)**	**95% CI**	**p**
**Pupil baseline**	Boundary slope	Block-Type	0.022	[-0.037 0.081]	0.474
		Trial-History	0.167	[0.132 0.202]	< 0.001
		Block-Type ×Trial-Type	0.038	[-0.024 0.099]	0.227

Supplementary Table 4. Main statistical effects on Accuracy and RT.

		Effect	Effect size (Odds Ratio)	95% CI	p
**Accuracy**	Generalized mixed-effects model	Block-Type × Trial-History × Accuracy Δ_{TH}	0.388	[0.280 0.539]	< 0.001
		Block-Type × Trial-History | LowACC-PC	0.924	[0.827 0.903]	0.002
		Block-Type × Trial-History | LowACC-PE	0.897	[0.512, 0.791]	< 0.001
		tVNS vs. SHAM |PC, LowACC-PC	0.964	[0.615, 0.913]	< 0.001
		PC vs. PE|SHAM, LowACC-PC	0.749	[0.715, 0.968]	0.003
		tVNS vs. SHAM |PE, LowACC-PE	0.335	[0.518 0.702]	< 0.001
		PC vs. PE|SHAM, LowACC-PE	0.621	[0.769 0.925]	< 0.001
		**Effect**	**Effect size (Estimate β)**	**95% CI**	**p**
**RT**	Linear mixed-effects model	Trial-History	64.75	[38.05 91.45]	< 0.001
		Block-Type × Trial-History	8.05	[-19.45 35.55]	0.566
		Block-Type × Trial-History × Accuracy Δ_{TH}	-75.41	[-347.89 197.06]	0.587

Supplementary Table 5. Effect sizes and 95% confidence intervals for DDM parameters.

	Parameter	Effect	F	p	Effect size (ω^2)
**Repeated measures ANOVA**	Drift rate	Trial-Type	18.960	< 0.001	0.045
		Block-Type	7.733	0.008	0.023
		Trial-Type×Block-Type	28.883	< 0.001	0.095
	Boundary intercept	Trial-Type	0.563	0.457	0.000
		Block-Type	0.047	0.828	0.000
		Trial-Type×Block-Type	1.686	0.200	0.003
	Boundary slope	Trial-Type	1.839	0.181	0.005
		Block-Type	0.918	0.343	0.000
		Trial-Type×Block-Type	5.132	0.028	0.021
	Non-decision time	Trial-Type	0.087	0.770	0.000
		Block-Type	1.186	0.281	0.0005
		Trial-Type×Block-Type	0.814	0.371	0.000
	**Parameter**	**Comparison**	**p**	**Effect size (Cohen's)**	**95% CI**
**Post Hoc analyses**	Drift rate	LowACC, tVNS vs LowACC, SHAM	< 0.001	0.870	[0.366 1.374]
		HighACC, SHAM vs LowACC, SHAM	< 0.001	-0.959	[-1.449 -0.469]
	Boundary slope	LowACC, tVNS vs LowACC, SHAM	0.204	-0.381	[-0.890 0.129]
		HighACC, SHAM vs LowACC, SHAM	1.000	0.087	[-0.445 0.619]

Supplementary Table 6. Stimulation intensities in tVNS and SHAM conditions.

Group	tVNS (mean ± SEM mA)	SHAM (mean ± SEM mA)
Early	1.70 ± 0.25	4.74 ± 0.70
Mid	1.38 ± 0.14	3.73 ± 0.39
Late	1.39 ± 0.11	3.19 ± 0.40
Overall	1.49 ± 0.10	3.88 ± 0.31

Data S1: Additional control analyses of pupil dynamics and behavioral effects.

Control analysis of pre-stimulation pupil dynamics

To assess whether the slight increase in pupil dilation observed before stimulation onset (Fig. 2A, middle panel) could reflect learned anticipation of the fixed stimulation timing, we performed an additional control analysis focusing on the 500ms preceding stimulation onset ([-0.5 0]s). Trials were separated into the first and second halves of the experiment, and the mean pupil dilation within this time window was analyzed using a linear mixed effects model:

$$\text{Pupil baseline} \sim \text{Session-Half} \times \text{Block-Type} \times \text{Group} + (1|\text{Participant})$$

Where Session-Half (first, second), Block-Type (tVNS, SHAM), and Group (Early, Mid, Late) were included as fixed factors, and Participant was included as a random intercept. However, the analysis revealed a non-significant tendency for pupil dilation during the [-0.5 0]s window to decrease rather than increase over the course of the experiment (main effect of Session-Half: $F = 3.225$, $p = 0.073$), with no interaction involving Block-Type ($F < 1.014$, $p > 0.363$) and no significant three-way interaction ($F = 0.052$, $p = 0.949$). Hence, this slight pre-stimulation pupil increase is unlikely to reflect learned anticipation of stimulation onset but may indicate a transient increase in attentional engagement at the beginning of the task, when the Fixation display with static dots appeared.

Relationship between tVNS-induced pupil dilation and behavioral improvement

To further evaluate the behavioral effects of tVNS could be explained by pupil-related optical factors, we examined the relationship between the magnitude of tVNS-induced pupil dilation (Pupil-AUC Δ_{BT}) and the magnitude of the behavioral effect of tVNS (Accuracy Δ_{BT}) across participants. Pupil-AUC Δ_{BT} was calculated as the difference in pupil AUC (area under the curve) between tVNS and SHAM conditions, and Accuracy Δ_{BT} was calculated as the difference in accuracy between tVNS and SHAM blocks. No significant correlation was observed when considering all trials ($r = 0.132$, $p = 0.308$; Supplementary Fig. 4, left panel). Importantly, the same result was obtained when restricting the analysis to LowACC trials, where the behavioral effect of tVNS was most pronounced ($r = 0.023$, $p = 0.861$; Supplementary Fig. 4, right panel). These findings indicate that individual differences in behavioral improvement were not systematically related to the magnitude of tVNS-induced pupil dilation, suggesting that the behavioral effects of tVNS were not directly determined by the magnitude of the pupil response itself.
